# Trends in hospital admissions during transition from paediatric to adult services for young people with learning disabilities or autism: Population-based cohort study

**DOI:** 10.1016/j.lanepe.2022.100531

**Published:** 2022-11-08

**Authors:** Ania Zylbersztejn, Philippa Anna Stilwell, Hannah Zhu, Viki Ainsworth, Janice Allister, Karen Horridge, Terence Stephenson, Linda Wijlaars, Ruth Gilbert, Michelle Heys, Pia Hardelid

**Affiliations:** a Population, Policy and Practice Research and Teaching Department, UCL Great Ormond Street Institute of Child Health, London, UK; b Community Child Health, Evelina Children's Hospital, London, UK; c Parent and Carer Advisory Group for Research, NIHR Great Ormond Street Hospital Biomedical Research Centre, London, UK; d NHS Lothian, Edinburgh, UK; e South Tyneside and Sunderland NHS Foundation Trust, UK; f Specialist Children's and Young People's Services, East London NHS Foundation Trust, London, UK

**Keywords:** Transition, Learning disability, Autism, Healthcare

## Abstract

**Background:**

Transition from paediatric to adult health care may disrupt continuity of care, and result in unmet health needs. We describe changes in planned and unplanned hospital admission rates before, during and after transition for young people with learning disability (LD), or autism spectrum disorders (ASD) indicated in hospital records, who are likely to have more complex health needs.

**Methods:**

We developed two mutually exclusive cohorts of young people with LD, and with ASD without LD, born between 1990 and 2001 in England using national hospital admission data. We determined the annual rate of change in planned and unplanned hospital admission rates before (age 10–15 years), during (16–18 years) and after (19–24 years) transition to adult care using multilevel negative binomial regression models, accounting for area-level deprivation, sex, birth year and presence of comorbidities.

**Findings:**

The cohorts included 51,291 young people with LD, and 46,270 autistic young people. Admission rates at ages 10–24 years old were higher for young people with LD (54 planned and 25 unplanned admissions per 100 person-years) than for autistic young people (17/100 and 16/100, respectively). For young people with LD, planned admission rates were highest and constant before transition (rate ratio [RR]: 0.99, 95% confidence interval [CI] 0.98–0.99), declined by 14% per year of age during (RR: 0.86, 95% CI: 0.85–0.88), and remained constant after transition (RR: 0.99, 95% CI: 0.99–1.00), mainly due to fewer admissions for non-surgical care, including respite care. Unplanned admission rates increased by 3% per year of age before (RR: 1.03, 95% CI: 1.02–1.03), remained constant during (RR: 1.01, 95% CI: 1.00–1.03) and increased by 3% per year after transition (RR: 1.03, 95% CI: 1.02–1.04). For autistic young people, planned admission rates increased before (RR: 1.06, 95% CI: 1.05–1.06), decreased during (RR: 0.95, 95% CI: 0.93–0.97), and increased after transition (RR: 1.05, 95%: 1.04–1.07). Unplanned admission rates increased most rapidly before (RR: 1.16, 95% CI: 1.15–1.17), remained constant during (RR: 1.01, 95% CI: 0.99–1.03), and increased moderately after transition (RR: 1.03, 95% CI: 1.02–1.04).

**Interpretation:**

Decreases in planned admission rates during transition were paralleled by small but consistent increases in unplanned admission rates with age for young people with LD and autistic young people. Decreases in non-surgical planned care during transition could reflect disruptions to continuity of planned/respite care or a shift towards provision of healthcare in primary care and community settings and non-hospital arrangements for respite care.

**Funding:**

National Institute for Health Research Policy Research Programme.

**Research in context**

**Evidence before this study**

We searched PubMed for studies examining health outcomes across transition from paediatric to adult health care in young people with learning/neurodevelopmental disabilities or autism spectrum disorders published between 1st January 2011 and 1st September 2021 in English. We used the search terms (autism OR autistic OR intellectual disabilit∗ OR learning disabilit∗ OR neurodevelopmental disabilit∗ OR neurodevelopmental delay) AND (transition∗ OR transfer∗) AND (to adult∗ OR from child∗ OR from pediatric OR from paediatric) AND (health service∗ OR healthcare OR health care OR admission∗ OR appointment OR visit) in titles and abstracts, checked reference lists of identified papers to find additional relevant studies and examined grey literature. We identified 10 qualitative and 12 quantitative studies.

Evidence from qualitative studies show that young people with learning disabilities or neurodevelopmental conditions, or autistic young people and their carers often have negative experiences of transition to adult health care. Families report longer waiting times, different eligibility criteria, lack of equivalent specialist services, poor information sharing between paediatric and adult services, and limited transition planning. A longitudinal study based on Italian administrative health records reported declines in the use of neuropsychiatric and rehabilitative services and an increase in use of mental health services in outpatient and community settings across ages 16–20 years old for autistic young people. According to the 2011 Scottish Census, young people with learning disabilities aged 19–24 years old report more mental health problems than those aged 13–18 years old. Two cross-sectional studies from the UK and US also showed that inpatient and outpatient care declined with age for young people with autism or neurodisabilities, while primary care visits increased after transition.

**Added value of this study**

This is the first whole population longitudinal cohort study to describe hospital admission trajectories before, during and after transition to adult care for young people with learning disabilities or autism spectrum disorders in England. We developed two national cohorts of 51,291 young people with learning disabilities and 46,270 autistic young people (with no learning disability indicated in hospital records), who were admitted to hospital at least once, likely capturing individuals with more complex health needs. We examined changes in planned and unplanned hospital admission rates before (aged 10–15 years old), during (16–18 years) and after (19–24 years) transition to adult care.

Planned admission rates declined during transition, mainly due to fewer admissions for respite care, and fewer and shorter (predominantly day case) non-surgical admissions. Unplanned admission rates did not change substantially with age (increased by 3% before, remained constant during and increased by 3% after transition) for young people with learning disabilities. For autistic young people, unplanned admission rates increased by 16% before, remained constant during and increased by 3% after transition, respectively. Increases were at least partially driven by admissions for non-specific symptoms, injury (due to self-harm) and mental health problems.

**Implications of all the available evidence**

Declines in rates of planned admissions at age 16–18 years old may reflect higher thresholds for planned hospital care in adult services. These trends could also be attributed to a shift towards provision of healthcare in primary care and community settings and alternative arrangements for respite care in non-hospital settings. Increases in unplanned admissions due to mental health problems and injury due to self-harm could indicate unmet mental health needs of young people entering adulthood. Conditions considered to be potentially preventable through treatment or better clinical management (such as constipation, reflux, or respiratory infections) also contributed to unplanned admissions across all ages, especially for young people with learning disabilities. Further research is needed to determine which interventions around transition to adult care would lead to best health outcomes for young people with learning disabilities or autism, and reduce mental health and other symptoms that result in unplanned admissions to hospital.

## Introduction

In the UK, around 2–3% of children and young people have a learning disability (LD) and 1–2% are autistic.^1^^,^^2^ Children and young people with LD or autism spectrum disorders (ASD) are more likely to report poor general health, mental health problems and have co-existing comorbidities (such as epilepsy, diabetes, obesity) than other children,^3^, ^4^, ^5^, ^6^, ^7^, ^8^ leading to more frequent interactions with healthcare.^9^^,^^10^

Transition into adulthood is a vulnerable period for all adolescents, with many simultaneous changes in their lives including leaving school, seeking first employment, and moving towards independent living. Young people with LD and/or ASD additionally face moving from paediatric to adult health and social care services, which can disrupt the continuity of care and impact on their health (see Research in Context). Paediatric care for children with complex health needs is often more holistic, with substantial support provided by specialist community and disability paediatricians and other child health providers outside and inside the hospital setting. Specialist adult care is more likely to be fragmented, with services being system or condition focussed, longer waiting times and higher thresholds for accessing services.^11^^,^^12^ Parents often report becoming advocates to help their young person navigate the transition to adult care.^11^, ^12^, ^13^, ^14^, ^15^

The NHS Long Term Plan set an ambition to ensure developmentally appropriate services for young people aged 16–25 years during transition to adult care and improved healthcare for children and young people with LD/ASD.^16^ Understanding how healthcare use changes during transition may inform interventions to better support young people during this sensitive time. The aim of this study was to describe changes in planned and unplanned admission rates before, during and after transition from paediatric to adult care for young people with an indication of LD or ASD in administrative hospital data in England.

## Methods

### Data sources

We used a de-identified extract of Hospital Episode Statistics (HES), an administrative dataset covering details of approximately 99% of all hospital care in England, linked to Office for National Statistics (ONS) mortality data, covering data from 1st January 1998 to 31st December 2018.^17^ Patients' hospital and mortality records can be tracked over time and between all NHS-funded hospital care using a study-specific patient identifier—the HESID—generated by the data provider. The HESID is generated through a multi-step algorithm based on combinations of patient's NHS number, date of birth, sex, postcode and local hospital identifier.^18^

Each hospital admission includes demographic information about the patient (such as age, sex), information about the admission (for example, dates of admission and discharge) and clinical information (diagnoses and procedures). Diagnoses in HES are coded using the International Classification of Diseases version 10 (ICD-10).^17^

### Study design

This is a descriptive longitudinal study of two cohorts of young people with LD or with ASD. Since LD and ASD are likely to co-exist (according to the 2011 Scottish Census about 20% of people with LD had co-existing ASD, and approximately 20% of autistic people had co-existing LD),^19^ we developed two mutually exclusive cohorts of young people whose hospital records indicated: (1) any type of LD or associated condition, or (2) ASD with no recording of co-existing LD (referred to as “autistic young people” or “young people with ASD” throughout the manuscript). This was to ensure that the findings for young people in each cohort are not driven by the same group of children with co-existing LD and ASD included in both. The cohort of autistic young people may, however, include individuals with LD not recorded in hospital records. The cohorts captured individuals born between 1st January 1990 and 31st December 2001 in England, who were admitted to hospital at least once. The cohorts therefore likely excluded more clinically mildly affected young people who did not require hospitalisation. Young people were followed from their 10th birthday, until 25th birthday, death or end of study period (31st December 2018) using administrative health records.

We classified young people at high risk of LD or with ASD if they had a relevant ICD-10 code recorded as any diagnosis during any hospital admission between 1998 and 2018, at any point before their 25th birthday (code lists are presented in Appendix Tables S1 and S3). We used all available hospital records to increase case ascertainment, as LD and ASD may not be recorded for every hospital admission, especially if hospital care is for other unrelated conditions. All individuals had between 2 and 10 years of historical data available before entering the study at age 10 years old, and between 7 and 14 years of follow-up data (Appendix Figure S1).

Specific ICD-10 codes for LD are likely to be under-recorded in hospital records,^20^ especially for children. We therefore derived a code list capturing a broad range of health conditions associated with LD in more than 30% of cases. Such an approach has been used to improve case ascertainment for studies of LD in adults using primary care data in England,^21^ although not all included individuals will have a learning disability. To indicate LD, we included: (i) ICD-10 codes explicitly indicating LD, (ii) codes indicating “high-risk” conditions where more than 75% of children are likely to have LD (such as Down syndrome), and (iii) codes indicating “associated” conditions where 30–75% of cases are likely to have LD (such as spastic quadriplegic cerebral palsy). The code list was derived through literature review followed by review by 5 experts who have experience working with young people with LD in primary care or community paediatrics. To indicate ASD we used ICD-10 code F84 and its subcodes excluding Rett syndrome. Further details on code list derivation and validation are described in Appendices 1.2–1.3. Young people with both LD and ASD were included in the LD cohort only.

### Outcomes

The main outcomes of interest were planned and unplanned hospital admission rates, defined as the number of admissions per 100 person-years at risk using years of follow-up for each child in the cohort as the denominator. Admissions were classified as planned/unplanned according to the admission method recorded in the first episode of care, excluding obstetric admissions which are expected to increase with age (see Appendix 1.4 for details). Admissions on the same day as a discharge (for example, hospital transfers) were treated as part of the same admission.^22^ Person-years at risk were calculated as time from the 10th birthday until end of follow-up (death, 25th birthday or 31st December 2018, whichever occurred first), excluding time spent in hospital during hospital admissions.

Secondary outcomes included the main reason for admission (according to the primary diagnosis) and length of stay.

### Exposure variable: transition from paediatric to adult care

Although age at transition is likely to vary between services and regions, it usually occurs between ages 16–18 years old (except for some very specialist care and early adopters of young person pathways covering care until 25th birthday).^23^ We therefore defined time before, during and after transition as ages 10–15, 16–18 and 19–24 years old, respectively,^24^ to reflect the typical configuration of transition services in England.

### Risk factors

We derived information about child's sex, year of birth (grouped as 1990–1993, 1994–1997, 1998–2001) and quintile of Index of Multiple Deprivation (IMD) score, an area-level deprivation measure derived for an average of 650 households in the child's area of residence.^17^ The score was allocated by the data provider based on the child's postcode. We used the IMD score recorded in hospital records in the year before entering the study (that is, aged 9 years old). For children who did not have a hospital admission in that year (and therefore would not have a recorded IMD score) we used mode of the scores recorded across all available hospital records.

We also derived an indicator of presence of other chronic conditions to account for complexity of health needs using a code list developed by Hardelid et al. The code list defines chronic conditions as health problems likely to require follow-up (for example, repeated hospital admission or specialist follow-up) for more than 1 year.^25^ We indicated chronic conditions from seven system groups listed in the code list (cancer/ blood disorders, chronic infections, respiratory conditions, metabolic/ endocrine/ digestive/ renal/ genitourinary conditions, musculoskeletal/ skin conditions, cardiovascular conditions, other non-specific conditions) if any of the relevant ICD-10 codes were recorded as any diagnosis (primary or secondary) during any hospital admission between 1998 and 2018 (excluding codes for LD or ASD). We then derived the number of chronic conditions from different system groups (categorised as none, one, two, three or more) as an approximate measure of complexity of health needs.

### Statistical analyses

#### Descriptive analyses

All analyses were run separately for cohorts of young people with LD and ASD (without LD). We estimated prevalence of LD and ASD in HES as the number of individuals in the study cohorts divided by the ONS mid-year population estimate for the number of children aged 10 years old in 2000–2011 (i.e. calendar years when study participants turned 10 years old).^26^

We described the distribution of risk factors in each cohort (overall and in the cohorts with complete information on all risk factors of interest).

#### Primary analyses

We estimated rate ratios from multilevel negative binomial regression models to determine the change in planned and unplanned admission rates before, during and after transition to adult care. We used admission counts as the outcome variable, the natural logarithm of person-time at risk as offset and age at admission as the main exposure. Models included three binary indicators (intercepts) and three linear splines (slopes) for each age category (10–15, 16–18 and 19–24 years old). Intercepts represent hospital admission rate per 100 person years at baseline (at ages 10, 16 and 19 years old, respectively, given baseline values of all other covariates). Slopes represent rate ratios for year-on-year change in hospital admission rates relative to the baseline age (i.e. how the admission rate changes with each year of age, assuming a linear trend). A priori we decided to include sex, deprivation, and number of chronic condition types as covariates in the models, to describe whether admission rates differ on average according to these basic characteristics. We also included birth year to account for possible cohort effects related to changes in coding depth and unequal length “look back” and “follow-up” periods available in HES data. Rate ratios for these covariates compare admission rates for children with different characteristics relative to the baseline group (for example, girls vs boys), given all other characteristics are equal. We allowed for a random effect by individual to account for correlation of child's outcomes (recurrent admissions) by age. We ran separate models for planned and unplanned admissions for each of the two cohorts. All analyses were based on complete case cohorts (i.e., with complete information on all risk factors of interest).

#### Secondary analyses

We derived the 10 most commonly recorded primary diagnoses (based on 3-character ICD-10 codes) at ages 10–15, 16–18 and 19–24 years. Based on this, we generated broader groups of common reasons for hospital admissions for young people with LD or ASD (ICD-10 codes listed in Appendix Tables S7 and S10) and we plotted trends in unadjusted admission rates by age for these groups. For planned admissions, we also separated admissions involving any surgical procedure and non-surgical admissions (criteria listed in Appendix Table S7). We derived mean and median length of stay, and the proportion of admissions by length of stay category (day cases, overnight stays, admissions lasting 1–6 days and 7+ days long) by age at admission.

#### Sensitivity analyses

We repeated the main analyses excluding children who died at any point during the study period to evaluate potential survivor bias (i.e. if young people with more complex health needs requiring more hospitalisations were more likely to die before transition). To explore possible cohort effects, we also repeated main analyses stratifying by year of birth category. Finally, we repeated main analyses for children with a specific diagnosis of LD or a “high-risk” condition (excluding children with only an indication of an “associated” condition).

### Role of the funding source

The funders of the study had no role in study design, data collection, data analysis, data interpretation, or writing of the report. The corresponding author had full access to all the data in the study and had final responsibility for the decision to submit for publication.

## Results

### Cohort characteristics

**Fig. 1:**
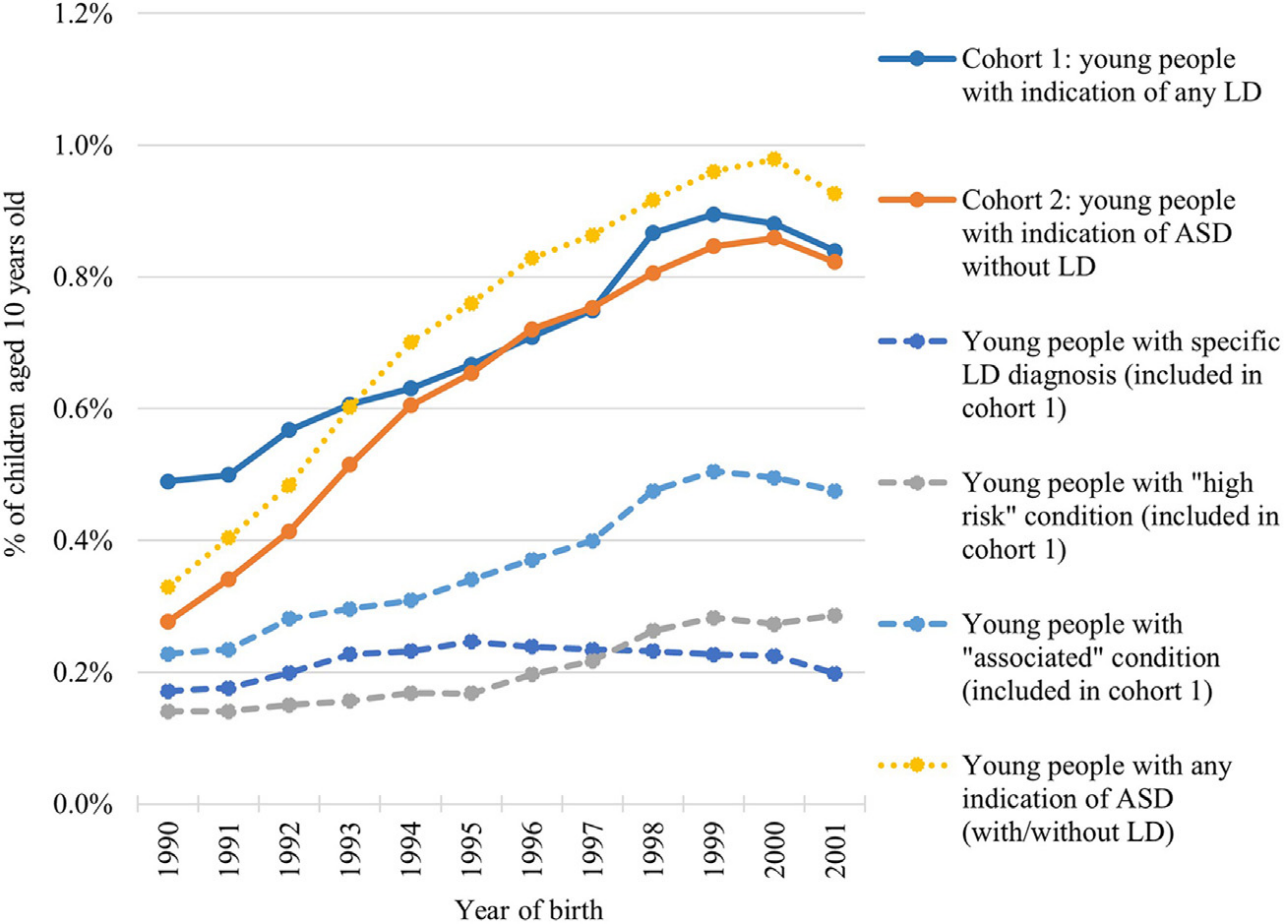
**Estimated incidence of learning disability (LD) and autism spectrum disorders (ASD) in children aged 10 years old in 2000–2011 in England, overall and by group of ICD-10 codes.** ASD = autistic spectrum disorders; ICD-10 = International Classification of Diseases version 10; LD = learning disability. Solid lines represent incidence of LD (cohort 1) and ASD without LD (cohort 2). Dashed lines represent incidence for 3 groups of conditions contributing to cohort 1: specific LD codes, “high risk” conditions and “associated” conditions (young people can have more than one condition). Dotted line represents incidence of ASD in all children (with or without LD) for comparison with nationally reported figures.

The first study cohort included 51,291 young people with LD, of whom 16,026 (31%) had an explicit LD diagnosis, 14,883 (29%) had an underlying “high risk” condition, 26,850 (52%) had an underlying “associated” condition and 6964 (14%) had an indication of both LD and ASD. The second cohort included 46,270 autistic young people (without LD). Corresponding population prevalence was 0.7% for each LD and ASD among children aged 10 years old in 2000–2011. The prevalence of both LD and ASD increased over time (Fig. 1), likely due to improved coding depth (Appendix Figures S2 and S3).

Complete information on all risk factors was available for 49,155 (96%) young people with LD and 45,743 (99%) autistic young people. Boys represented 55% of young people with LD and 74% with ASD (Table 1). 73% of young people with LD and half of autistic young people had at least one co-existing chronic condition. 29% and 25% of young people with LD and ASD, respectively, lived in the most deprived 20% of areas compared to 15–17% in the least deprived areas, reflecting England's unequal distribution of births by area-level deprivation.^27^

**Table 1:** Cohort characteristics for young people with learning disabilities or autism spectrum disorders (overall and in cohorts with complete information on all risk factors).

Risk factors	Young people with learning disability		Autistic young people (with no recorded learning disabilities)	
Total	Whole cohort n = 51,291	Complete case n = 49,155 (96%)	Whole cohort n = 46,270	Complete case n = 45,743 (99%)
**Sex**				
Male	27,694 (54%)	26,826 (55%)	34,283 (74%)	33,911 (74%)
Female	23,169 (45%)	22,329 (45%)	11,902 (26%)	11,832 (26%)
*Missing*	428 (1%)		85 (0.2%)	
**IMD Quintile**				
Q1: most deprived	14,260 (28%)	14,122 (29%)	11,477 (25%)	11,467 (25%)
Q2	10,510 (20%)	10,399 (21%)	9994 (22%)	9957 (22%)
Q3	9011 (18%)	8956 (18%)	8881 (19%)	8859 (19%)
Q4	8166 (16%)	8098 (16%)	7842 (17%)	7833 (17%)
Q5: least deprived	7631 (15%)	7580 (15%)	7634 (16%)	7627 (17%)
*Missing*	1713 (3%)		442 (1%)	
**Presence of different groups of chronic conditions (out of 7 categories)**				
None	14,565 (28%)	13,280 (27%)	23,578 (51%)	23,220 (51%)
1	13,666 (27%)	13,019 (26%)	13,935 (30%)	13,807 (30%)
2	9747 (19%)	9590 (20%)	5785 (13%)	5751 (13%)
3+	13,313 (26%)	13,266 (27%)	2972 (6%)	2965 (6%)
**Year of birth**				
1998–2001	20,358 (40%)	18,864 (38%)	19,494 (42%)	19,158 (42%)
1994–1997	16,913 (33%)	16,586 (34%)	16,765 (36%)	16,637 (36%)
1990–1993	14,020 (27%)	13,705 (28%)	10,011 (22%)	9948 (22%)

IMD = Index of Multiple Deprivation.

### Planned admission rates

#### Primary analyses

**Fig. 2:**
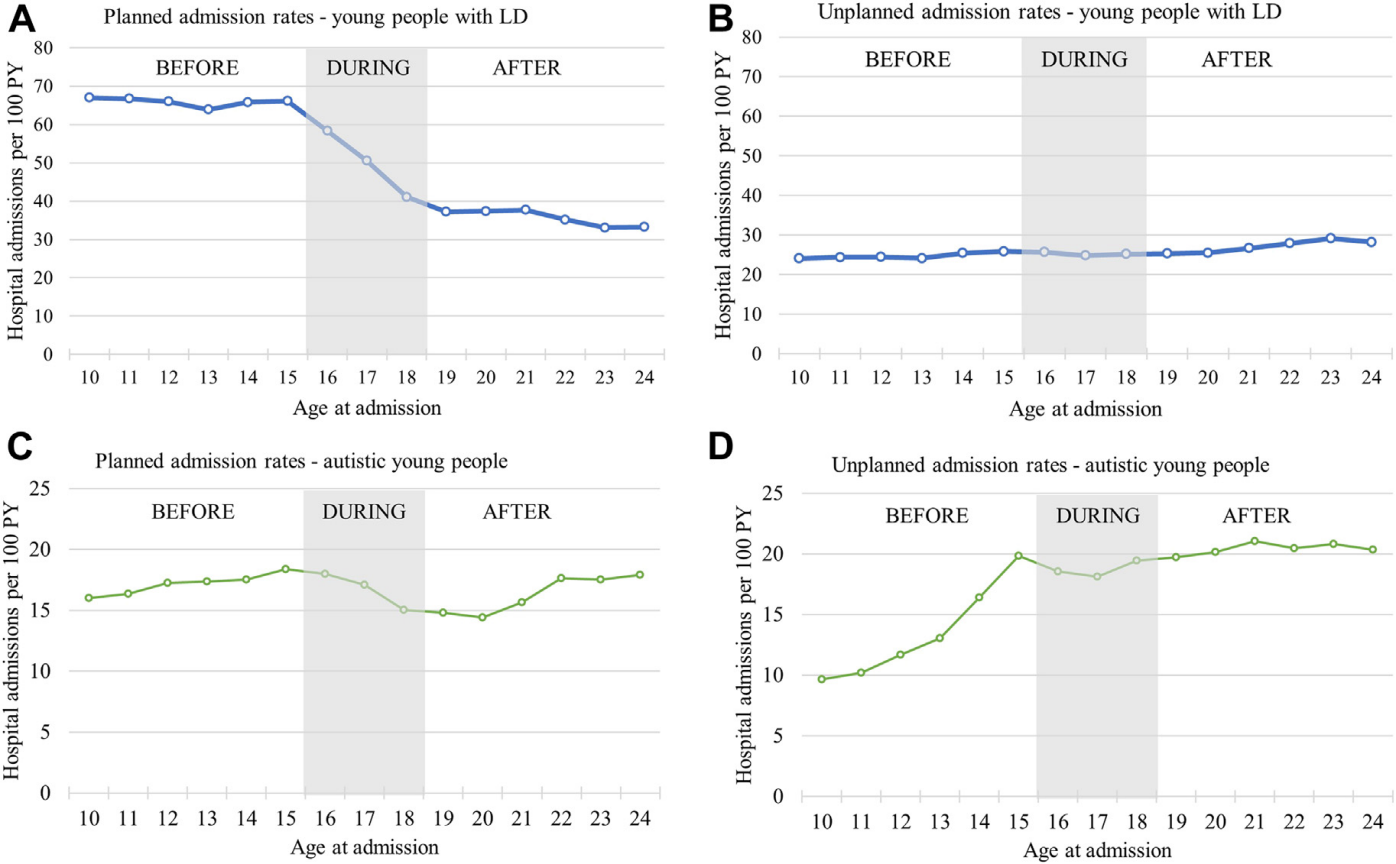
**Crude hospital admission rates for young people with learning disabilities (LD) or autism spectrum disorders (ASD) before, during and after transition to adult services.** PY = person years at risk. Note different scales of y-axes for young people with LD and ASD.

Young people with LD had 54 planned admissions per 100 person-years aged 10–24 years old (315,261 admissions in total, Fig. 2A). 72% had at least one planned admission during the study period, 40% had more than three. Planned admission rates were highest and constant before transition (rate ratio [RR]: 0.99, 95% confidence interval: 0.98–0.99), declined by 14% per year of age during transition (RR: 0.86, 0.85–0.88), and remained constant after transition (RR: 0.99, 0.99–1.00, Table 2).

**Table 2:** Adjusted hospital admission rates and rate ratios (95% confidence intervals) for planned and unplanned admissions for young people with learning disabilities (LD) and autistic young people derived from multilevel negative binomial models.

	Young people with LD		Autistic young people (without recorded LD)	
	Planned admissions	Unplanned admissions	Planned admissions	Unplanned admissions
**Admission rates at baseline for each age group per 100 person-years (intercept)**				
Age 10	6.7 (6.4, 7.1)	2.3 (2.2, 2.5)	5.3 (5.1, 5.6)	4.0 (3.8, 4.2)
Age 16	7.0 (6.6, 7.4)	2.7 (2.5, 2.9)	7.0 (6.6, 7.4)	7.9 (7.4, 8.4)
Age 19	3.9 (3.6, 4.1)	2.8 (2.6, 2.9)	5.5 (5.2, 5.9)	8.4 (8.0, 8.8)
**All estimates below are rate ratios**				
**Change in admission rate per year by age group (slope)**				
Age 10–15	0.99 (0.98, 0.99)	1.03 (1.02, 1.03)	1.06 (1.05, 1.06)	1.16 (1.15, 1.17)
Age 16–18	0.86 (0.85, 0.88)	1.01 (1.00, 1.03)	0.95 (0.93, 0.97)	1.01 (0.99, 1.03)
Aged 19–24	0.99 (0.99, 1.00)	1.03 (1.02, 1.04)	1.05 (1.04, 1.07)	1.03 (1.02, 1.04)
**Sex**				
Male (baseline)	1	1	1	1
Female	0.89 (0.87, 0.91)	1.03 (1.00, 1.06)	0.93 (0.90, 0.96)	1.56 (1.52, 1.60)
**IMD Quintile**				
Q1: Most deprived 20%	0.81 (0.78, 0.84)	1.25 (1.19, 1.31)	0.93 (0.89, 0.97)	1.03 (0.99, 1.07)
Q2	0.88 (0.84, 0.92)	1.14 (1.09, 1.20)	0.93 (0.89, 0.98)	1.01 (0.97, 1.05)
Q3	0.94 (0.90, 0.99)	1.08 (1.02, 1.14)	0.95 (0.91, 1.00)	1.03 (0.99, 1.08)
Q4	0.98 (0.93, 1.02)	1.04 (0.99, 1.10)	0.97 (0.93, 1.02)	0.99 (0.94, 1.03)
Q5: Least deprived 20%	1	1	1	1
**Year of birth**				
1998–2001	1	1	1	1
1994–1997	1.22 (1.18, 1.26)	1.25 (1.20, 1.29)	0.87 (0.85, 0.90)	0.78 (0.76, 0.80)
1990–1993	1.52 (1.47, 1.58)	1.51 (1.46, 1.57)	0.90 (0.87, 0.93)	0.77 (0.74, 0.79)
**Presence of different groups of chronic conditions** (out of 7)				
None (baseline)	1	1	1	1
1	1.92 (1.84, 1.99)	1.66 (1.59, 1.74)	1.56 (1.51, 1.61)	1.54 (1.50, 1.59)
2	3.32 (3.19, 3.46)	2.80 (2.67, 2.93)	2.70 (2.60, 2.81)	2.29 (2.21, 2.38)
3+	9.25 (8.90, 9.60)	8.46 (8.11, 8.82)	6.26 (5.98, 6.57)	4.42 (4.22, 4.63)

IMD = Index of Multiple Deprivation.

All estimates are mutually adjusted for covariates listed in the table.

Autistic young people had 17 planned admissions per 100 person-years (90,586 admissions in total); 61% had at least one planned admission during the study period, and 16% had more than three. Admission rates increased by 6% per year of age before transition (RR: 1.06, 1.05–1.06), declined by 5% per year of age during transition (RR: 0.95, 0.93–0.97) and increased by 5% per year of age after transition (RR: 1.05, 1.04–1.07).

In both cohorts, planned admission rates were lower for girls than boys, and rates were lower for young people living in more deprived areas. Children with multiple chronic conditions had higher planned admission rates than children with no co-existing chronic conditions.

#### Secondary analyses

**Fig. 3:**
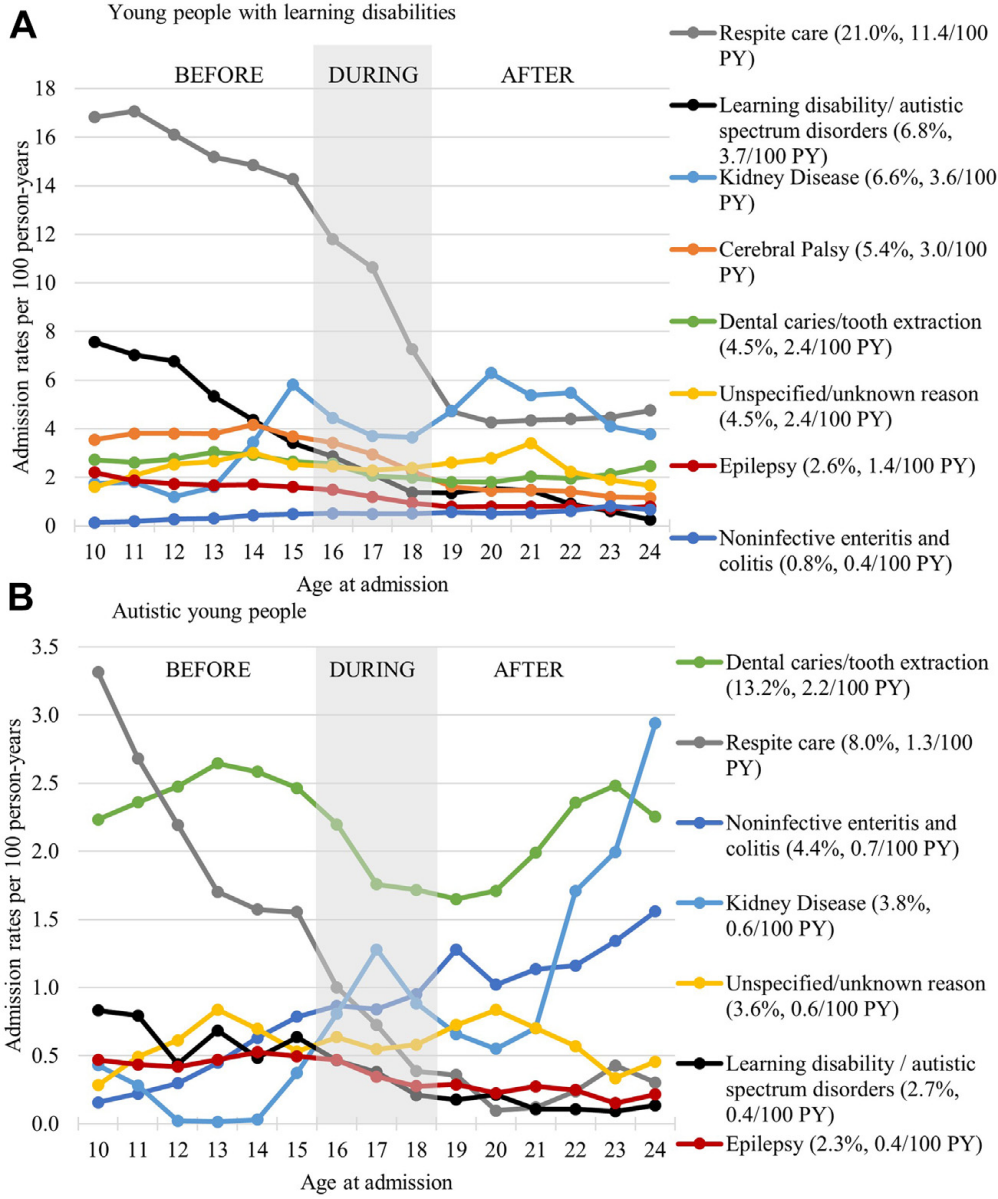
**Crude planned admission rates by the main reason for admission for young people with (A) learning disabilities and (B) autistic young people (without learning disabilities) before, during and after transition to adult services.** We present trends for the two cohorts in parallel focussing on selected groups of the most common reasons for planned hospital admissions. In brackets we present crude admission rate per 100 person-years (PY) and the proportion of all admissions due to each cause. Note different scales of y-axes for young people with learning disabilities and autistic young people.

The most common reasons for planned admission for young people with LD were respite care (11.4 admissions per 100 person-years, accounting for 21% of admissions), interventions related to learning disabilities/autism (3.7/100, 6.8% of admissions). Admission rates due to these causes declined with age (Fig. 3A). Most common reasons for planned admissions for autistic young people were tooth extractions (2.2/100 person-years, 13% of admissions) and respite care (1.3/100 person-years, 8% of admissions, Fig. 3B). Non-surgical admission rates declined in both cohorts (Appendix Figure S4).

Planned admissions became shorter after transition to adult care. The proportion of day cases increased from 44% to 65% of admissions at ages 10–15 and 19–24 for young people with LD, and from 66% to 80% for autistic young people, respectively, while the proportion of planned overnight stays declined (Appendix Tables S11 and S12).

### Unplanned admission rates

#### Primary analyses

Young people with LD had 25 unplanned admissions per 100 person-years between the ages of 10–24 years (147,146 unplanned admissions overall, Fig. 2C); 29% had more than three while 40% did not have any unplanned admissions during the study period. Unplanned admission rates increased marginally with age, by 3% per year of age before transition (RR: 1.03, 1.02–1.03), remained constant during transition (RR: 1.01, 1.00–1.03) and increased moderately by 3% per year of age after transition to adult care (RR: 1.03, 1.02–1.04, Table 2). Unplanned admission rates increased with level of deprivation, with children living in most deprived areas having 25% higher unplanned admission rates than children in the least deprived areas. Having multiple chronic conditions was associated with higher unplanned admission rates.

Autistic young people had 16 unplanned admissions per 100 person-years between the ages of 10–24 years (89,112 admissions in total, Fig. 2D). 21% of young people had more than 3 admissions during the study period, while 35% of children had no unplanned admissions. Admission rates increased most rapidly by 16% per year of age before transition (RR: 1.16, 1.15–1.17), remained constant during transition (RR: 1.01, 0.99–1.03), and increased moderately by 3% per year of age after transition (RR: 1.03, 1.02–1.04). Girls had 56% higher unplanned admission rates than boys. There was no effect of IMD quintile on emergency admissions in young people with ASD.

#### Secondary analyses

**Fig. 4:**
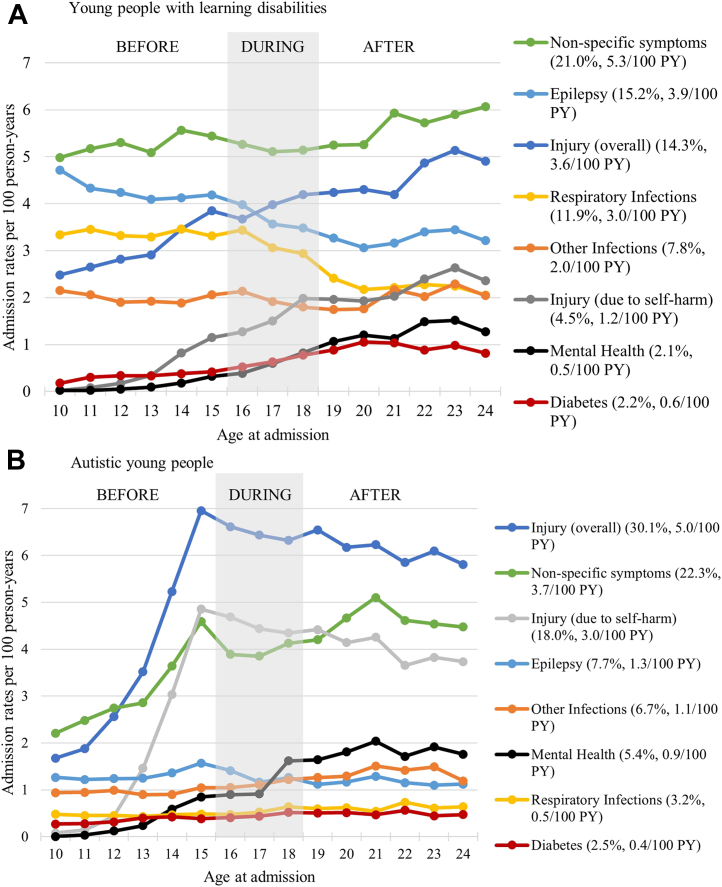
**Crude unplanned admission rates by the main reason for admission for young people with (A) learning disabilities and (B) autistic young people (with no learning disabilities).** We present trends for the two cohorts in parallel focussing on selected groups of the most common reasons for planned hospital admissions. In brackets we present crude admission rate per 100 person-years (PY) and the proportion of all admissions due to each cause.

The most common reasons for unplanned admissions in young people with LD were non-specific symptoms (such as headache, nausea and vomiting, or abdominal pain, accounting for 21% of all unplanned admissions and on average 5.3 admissions per 100 person-years), epilepsy (15%, 3.9/100 person-years), injury (accidental or non-accidental, 14%, 3.6/100 person-years) and respiratory infections (12%, 3.0/100 person-years, Fig. 4A). For autistic young people, 30% of all unplanned admissions were due to injuries (5 admissions per 100 person-years, over half of which were due to self-harm), 22% were due to non-specific symptoms (3.7/100 person-years), and 8% were due to epilepsy (1.3/100 person-years). In both cohorts, admissions due to non-specific symptoms and injury due to self-harm increased with age, most rapidly for young people with ASD aged 10–15 years old. Mental health problems accounted for only 2% and 5% of all unplanned admissions for young people with LD and ASD, respectively, but mental health-related unplanned admission rates consistently increased with age.

Unplanned admissions became longer with age. This was due to an increase in the proportion of admissions lasting more than a week (from 13% to 19% at ages 10–15 and 19–24 for young people with LD, and from 7% to 15% for autistic young people, respectively) and a decline in the proportion of day cases and overnight stays (from 59% to 50% at ages 10–15 and 19–24 for young people with LD and from 69% to 60% for autistic young people, respectively, Appendix Tables S13 and S14).

### Sensitivity analyses

Results from sensitivity analyses excluding children who died, and splitting the data by year of birth showed consistent results for planned and unplanned admissions (Appendix Tables S15–S19, respectively). This suggest that the results are not likely to be affected by possible cohort effects or survivor bias. Results focussing on young people with a specific learning disability diagnosis or a diagnosis of a “high-risk” condition (that is, excluding young people with only “associated” conditions) were also consistent (Appendix Table S20).

## Discussion

### Key findings

Planned admission rates declined by 14% per year of age during transition for young people with LD and by 5% for autistic young people; this was driven by a decline in non-surgical admissions, in particular for respite care. Unplanned admission rates showed a moderate increase with age for young people with LD before and after transition (by 3% per year of age in each age group), and a more rapid increase for autistic young people (by 16% before, and by 3% per year of age after transition). The most common reasons for unplanned admissions were non-specific symptoms, injury and epilepsy.

### Strengths and limitations

This is the first whole population longitudinal cohort study to describe hospital admission trajectories before, during and after transition to adult care for young people with LD or ASD in England. We used a national administrative health database covering details of 99% of hospital admissions in England. Availability of hospital records over 20 years enabled us to track hospital admission trajectories from childhood into adulthood.

Our estimated prevalence was lower than national estimates based on school census data (0.7% for each LD and ASD in our study, compared to 2.5% and 1.7%, respectively^1^^,^^2^), and over 70% of children with LD and 50% of autistic children had at least one additional chronic condition. These differences reflect the fact that we included only individuals admitted to hospital at least once, who likely have more complex health needs, omitting young people with mild LD or ASD and no comorbidities. Our results are therefore generalisable only to children with LD or ASD and complex health needs.

One limitation is that specific diagnostic codes indicating LD/ASD are likely to be under-recorded in hospital records,^20^ especially for children. To increase case ascertainment in absence of a LD/ASD register, we used a broad diagnostic code list capturing conditions associated with LD in more than 30% of cases, reviewed by 5 clinicians who work with children with LD/ASD. Such approach has been applied in studies based on primary care data in England.^21^ However, not all young people included in LD cohort will definitely have LD (for example, approximately 50% of children with cerebral palsy have associated LD^28^). Further work exploring health outcomes for a wider cohort of young people with LD or ASD requires disease registry data, which would enable identification of all individuals with learning disability and autism and examination of health outcomes of young people with both LD and ASD. However, such data are not available in England. Instead, further work could focus on using primary care data, as general practitioners have been asked to keep a register of all patients aged over 14 years who have learning disabilities and to offer them annual health checks since 2014.^1^ Young people with LD/ASD could also be indicated using School Census information from National Pupil Database, where LD and ASD are indicated as types of need for additional educational provisions.^29^ These data have now been linked to HES in the ECHILD database.^30^

The second limitation is that our analyses focussed on hospital admissions. We were not able to examine patterns of interactions with outpatient, primary and community care settings, where most routine care for young people with LD or ASD is delivered. Further research to examine possible gaps in service provision and geographical variation in local configuration of services could be carried out using primary care data, community health services data and HES outpatient records. However, improvements to quality of HES outpatient data are needed. First, diagnoses are missing for most outpatient appointments. Second, coverage is likely to vary geographically between specialties (for example, community paediatrics is likely to be underreported in HES Outpatient Data). We therefore focused on hospital admissions, which likely indicate young people with more severe LD or ASD or with comorbidities.

The third limitation is that we could not compare healthcare use in children with LD and ASD to the general population. HES data only capture children who were admitted to hospital at least once, therefore information about follow-up and risk factors is not available for children with no hospital admissions. A cross-sectional study of healthcare use in all young people in England in 2009 showed that unplanned admission rates increased from 3.2 per 100 person-years at age 10–14 years to 5.2/100 at age 20–24 years; planned admission rates also increased from 2.9/100 to 4.9/100 respectively.^31^ In our study, the corresponding planned and unplanned admission rates were 4–17 times higher for young people with LD and 3 times higher for young people with ASD. Further work focussing on pupils attending school using the ECHILD database^30^ or children registered with a general practitioner using primary care data would enable a comparison with the general population.

Finally, our models required a number of assumptions. First, we assumed a linear trend in admission rates at ages 10–15, 16–18 and 19–24 years old. This simple approach enabled direct interpretation of coefficients and visual assessment of crude rates suggested this assumption is reasonable. However, we acknowledge that it might attenuate some of the year-on-year changes in admission rates. Second, our models assume that admission rate ratios for included covariates are constant with age, averaging out any differences arising with age (for example, between sexes, IMD quintiles and children with multiple chronic conditions). Third, we used the number of chronic conditions from different system groups as a simplified measure of complexity of health needs. Further studies examining the contribution of different chronic conditions are needed. Future studies should also account for changes in IMD scores over time (as IMD was only recorded when a child was admitted to hospital). This would be possible using the ECHILD database, where free school meal eligibility and area of residence are recorded in each school year.^30^

### Interpretation

#### Planned admission rates

Non-surgical hospital admissions for planned care (in particular, for respite care) declined during transition for young people with LD and ASD, with more day cases and fewer overnight stays within adult services. These findings might reflect higher thresholds for non-surgical planned hospital admissions or lack of provision of respite care in adult care. These trends may also partially reflect better management of associated health problems in adulthood (for example epilepsy, for which we observed declines in unplanned admission rates with age). Observed trends could also reflect a shift towards provision of care in other settings. A cross-sectional study of young people with neurodisabilities in England found that declines in hospital admissions and outpatient appointments around the age of transition were paralleled by increases in primary care consultation rates.^10^ In our study, cross-sectional analyses of HES outpatient data for young people with LD or ASD in 2014–2018 indicated similar declines (analyses not shown). Average number of attended appointments per child declined from 3.9 appointments aged 10–15, to 3.0 aged 16–18 and to 2.2 aged 18–24 years for young people with LD (Appendix Figure S6). Corresponding figures for autistic young people were 2.3, 1.8 and 1.4, respectively. Further research using longitudinal liked data from primary, community and secondary care is needed to determine if and how these changes in provision impact on young people's health.

#### Unplanned admission rates

Unplanned admission rates increased with age for both cohorts. The most common reasons for unplanned admissions included non-specific symptoms (predominantly due to abdominal pain, including constipation and reflux), epilepsy and for young people with LD, respiratory infections. Some of these unplanned admissions may be preventable through better clinical management or treatment (for example, early antibiotic treatment for respiratory infections, better management of epilepsy),^32^ and could be affected by inequalities in access to healthcare and health literacy. Since 2014, general practitioners have been incentivised to offer annual health checks for people with LD aged 14 and over.^1^ One study showed that these health checks were associated with reductions in unplanned admissions due to such potentially preventable conditions in adults.^32^ Further work is needed to determine impact of this scheme on potentially preventable unplanned admissions in children with LD, and assessing whether access to care is equitable.

We also observed increases in unplanned admissions due to self-harm injuries and mental health problems, especially for autistic young people. Although these trends are consistent with the general population,^24^ young people with LD and/or ASD have higher rates of mental health problems than their peers across all ages.^7^^,^^8^ We cannot determine whether the increase in unplanned admissions would be reduced if planned hospital contacts or community-based specialist care continued at rates provided as part of paediatric and children and young people's mental health services. However, many young people with neurodevelopmental disabilities and their carers worry about meeting eligibility criteria for adult mental health services.^12^ Joint transition planning between child and adult mental health services, primary and community care might help to reduce the need for unplanned hospital care for young people with LD/ASD.

#### Transition to adult care

The prevalence of LD and/or ASD among young adults is growing, partially due to increased survival of young people with complex conditions. Healthcare support for these young people in adulthood therefore needs to address their complex health and social needs. Evidence is limited on effective interventions to improve transition to adult care.^33^^,^^34^ Since 2016, national guidelines in the UK recommend that transition planning should start at age 13/14 years old at the latest, be developmentally appropriate, and co-ordinated by a named worker.^35^ Further work is needed to determine how interactions with healthcare have changed for young people since the introduction of these guidelines and the impact of health service models designed to operationalise these guidelines. Appropriate involvement of parents or carers, promoting self-efficacy and meeting the adult services team before transition are associated with better satisfaction with services, wellbeing and gaining autonomy in managing health for young people with long-term conditions.^36^ The NHS Long Term Plan also sets an ambition to ensure that by 2023/24 young people and young people with a LD/ASD with the most complex needs will have a designated keyworker to support coordination of care around transition.^16^

Undoubtedly, family- and young person-centred approaches are needed for young people with LD and ASD, recognising that families carry the main caring responsibility for young people with long-term conditions (informal care provided by families accounts for 66% of total care costs for young people during transition).^37^ Caring for a young person with LD or ASD can be a major source of stress and contribute to adverse mental and physical health outcomes for the carers,^38^^,^^39^ therefore provision of appropriate respite care is important for parents’ wellbeing. We found that respite care accounted for a fifth of planned admissions for young people with LD and rates reduced by 70% after transition. Further work is needed to determine whether this decline represents a shift towards provision of respite care in community rather than hospital setting or if it indicates unmet needs, and if so, how best to address these unmet needs.

### Conclusions

Young people with LD/ASD indicated in their hospital records had fewer and shorter planned admissions and more and longer unplanned admissions after transition. The decline in planned care during transition could reflect higher thresholds for planned care in adult services, better management of health conditions or a shift towards provision of healthcare in other settings (for example residential care placements). Increases in unplanned admissions due to mental health problems and self-harm could indicate unmet mental health needs of young people entering adulthood. Transition planning involving child and adult secondary, community and primary care services is needed to ensure best health outcomes for young people with LD/ASD. Our study was limited to young people with LD/ASD recorded in hospital record, capturing population with complex health needs. Further research using data on all children with LD/ASD and a reference population is therefore needed to validate our findings and determine which interventions would improve health outcomes for young people with LD or autism more broadly and lead to fewer unplanned admissions.

## Contributors

The study was designed by AZ and PH. AZ carried out literature review to develop the code list. PAS HZ, JA, KH, TS, MH reviewed preliminary code list. LW prepared initial data extract. AZ developed the cohort, analyzed the data and wrote the first draft of the manuscript. All authors interpreted the data and contributed to subsequent drafts of the manuscript. All authors have seen and approved the final version.

## Data sharing statement

The authors do not have permission to share patient-level HES data. Source data for England can be accessed by researchers applying to the NHS Digital. Code lists and Stata code used for this project are freely available at https://github.com/UCL-CHIG/.

## Declaration of interests

The authors declare that they have no conflict of interest.
