# Supplementary material for: Trends in hospital admissions during transition from paediatric to adult services for young people with learning disabilities or autism: Population-based cohort study
Source: Lancet Reg Health Eur. 2022 Nov 8;24:100531. doi: 10.1016/j.lanepe.2022.100531 (PMC9649375; doi:10.1016/j.lanepe.2022.100531)
Supplement: Supplementary Materials [file mmc1.docx]

# Definitions

## Data available for the study

We developed two non-overlapping cohorts of young people with learning disabilities (LD) and autism spectrum disorders (ASD). The cohorts captured young people with LD or ASD who had an appropriate ICD-10 code recorded as any diagnosis (primary or secondary) during any hospital admission between 1^st^ January 1998 and 31^st^ December 2018 before their 25^th^ birthday. All individuals had between 2 and 10 years of historical data available before entering the study at age 10, and between 7 and 14 years of follow-up data (Appendix Figure 1). Median follow-up time was 12.0 years (interquartile range: 9.5-14.8 years) for children with LD and 12.0 (ICR: 9.5-14.6 years) for young people with ASD.

*Appendix Figure 1 - Age at admission for young people in the cohort by year of birth and year of admission*

|  |  | Age | | | | | | | | | | | | | | | | | | | | | | | | |
| --- | --- | --- | --- | --- | --- | --- | --- | --- | --- | --- | --- | --- | --- | --- | --- | --- | --- | --- | --- | --- | --- | --- | --- | --- | --- | --- |
|  |  | 0 | 1 | 2 | 3 | 4 | 5 | 6 | 7 | 8 | 9 | **10** | 11 | 12 | 13 | 14 | 15 | 16 | 17 | 18 | 19 | 20 | 21 | 22 | 23 | 24 |
| Year of birth | 1990 |  |  | Not available (before 1998) |  |  |  |  |  |  |  |  |  |  |  |  |  |  |  |  |  |  |  |  |  |  |
|  | 1991 |  |  |  |  |  |  |  |  |  |  |  |  |  |  |  |  |  |  |  |  |  |  |  |  |  |
|  | 1992 |  |  |  |  |  |  |  |  |  |  |  |  |  |  |  |  |  |  |  |  |  |  |  |  |  |
|  | 1993 |  |  |  |  |  |  |  |  |  |  |  |  |  |  |  |  |  |  |  |  |  |  |  |  |  |
|  | 1994 |  |  |  |  |  |  |  |  |  |  |  |  |  |  |  |  |  |  |  |  |  |  |  |  |  |
|  | 1995 |  |  |  |  |  |  |  |  |  |  |  |  |  |  |  |  |  |  |  |  |  |  |  |  |  |
|  | 1996 |  |  |  | Additional data used for case identification |  |  |  |  |  |  |  |  |  |  |  |  |  |  |  |  |  |  |  |  |  |
|  | 1997 |  |  |  |  |  |  |  |  |  |  |  |  |  |  |  |  |  |  | Follow-up records |  |  |  |  |  |  |
|  | 1998 |  |  |  |  |  |  |  |  |  |  |  |  |  |  |  |  |  |  |  |  |  |  |  |  |  |
|  | 1999 |  |  |  |  |  |  |  |  |  |  |  |  |  |  |  |  |  |  |  |  |  |  |  |  |  |
|  | 2000 |  |  |  |  |  |  |  |  |  |  |  |  |  |  |  |  |  |  |  |  |  |  |  |  |  |
|  | 2001 |  |  |  |  |  |  |  |  |  |  |  |  |  |  |  |  |  |  |  |  |  |  |  |  |  |

## Code list for young people with Learning Disabilities

### Overview of included codes

While there are specific ICD-10 codes to indicate LD, they are likely to be under-recorded. We therefore aimed to derive a broader code list, capturing LD or conditions associated with LD in more than 30% of cases. Such approach has been used to improve case ascertainment for studies of learning disabilities in adults using primary care data, although not all indicated individuals will have a learning disability.^1^

We defined learning disability according to three core criteria (based on definition from Quality and Outcomes Framework (QOF) used in UK primary care^2^):

- Lower intellectual ability (usually defined as an Intelligence Quotient of less than 70) AND
- Significant impairment of social or adaptive functioning; AND
- Onset in childhood.

We carried out a literature review to identify ICD-10 codes used to indicate learning disability or conditions likely to lead to learning disability in more than 30% of cases. The preliminary code list was reviewed by 5 expert clinicians (who have experience working with children with LD/ASD in primary care or community paediatrics) to indicate conditions relevant to young people. We then combined information from experts and included codes where the majority of experts agreed. We also included a number of non-specific codes (such as “Other congenital malformations, not elsewhere classified”) after examining incidence of specific LD codes in individuals with these conditions.

The final code list covered three groups of codes (referred to as “coding clusters” throughout the appendix):

- **Specific learning disability diagnosis** codes from ICD-10 chapter F7
- **“High risk” conditions:** condition where >75% of young people are likely to have a learning disability (such as Down syndrome, Edwards, Patau, Fragile X in boys – see Appendix Table 2)
- **“Associated” conditions:** conditions where between 30-75% of young people are likely to have a learning disability (such cerebral palsy – see Appendix Table 2)

Appendix Table 1 – ICD-10 codes used to indicate young people with learning disability in HES

| **ICD-10 code** | **Description** | **Coding cluster** |
| --- | --- | --- |
| F70 | Mild mental retardation | Exact LD diagnoses |
| F71 | Moderate mental retardation |  |
| F72 | Severe mental retardation |  |
| F73 | Profound mental retardation |  |
| F78 | Other mental retardation |  |
| F79 | Unspecified mental retardation |  |
| Q90 | Down syndrome | “High risk” conditions where ≥75% of young people are likely to have LD |
| Q91 | Edwards syndrome and Patau syndrome |  |
| Q92.0 | Whole chromosome trisomy, meiotic nondisjunction |  |
| Q92.1 | Whole chromosome trisomy, mosaicism (mitotic nondisjunction) |  |
| Q92.2 | Major partial trisomy |  |
| Q92.3 | Minor partial trisomy |  |
| Q92.4 | Duplications seen only at prometaphase |  |
| Q92.5 | Duplications with other complex rearrangements |  |
| Q92.7 | Triploidy and polyploidy |  |
| Q92.8 | Other specified trisomies and partial trisomies of autosomes |  |
| Q92.9 | Trisomy and partial trisomy of autosomes, unspecified |  |
| Q93 | Monosomies and deletions from the autosomes, not elsewhere classified |  |
| Q99.8 | Other specified chromosome abnormalities |  |
| Q99.9 | Chromosomal abnormality, unspecified |  |
| Q89.9 | Other congenital malformations, not elsewhere classified |  |
| Q99.2 | Fragile X chromosome *(in boys)* |  |
| Q00 | Anencephaly and similar malformations |  |
| Q01 | Encephalocele |  |
| F84.2 | Rett syndrome |  |
| E00 | Congenital iodine-deficiency syndrome |  |
| E75 | Disorders of sphingolipid metabolism and other lipid storage disorders |  |
| E79.1 | Lesch‐Nyhan syndrome |  |
| E83.0 | Disorders of copper metabolism (Menkes disease) |  |
| E76 | Disorders of glycosaminoglycan metabolism | “Associated” conditions where 30-75% of young people are likely to have LD |
| E77 | Disorders of glycoprotein metabolism |  |
| E88.8 | Other specified metabolic disorders |  |
| G80.0 | Spastic quadriplegic cerebral palsy |  |
| Q86.0 | Foetal alcohol syndrome (FAS) |  |
| Q86.1 | Foetal hydantoin syndrome |  |
| Q86.2 | Dysmorphism due to warfarin |  |
| Q86.8 | Other congenital malformation syndromes due to known exogenous causes |  |
| Q02 | Microcephaly |  |
| Q03 | Congenital hydrocephalus |  |
| Q04 | Other congenital malformations of brain |  |
| Q85.1 | Tuberous sclerosis |  |
| Q85.8 | Other phakomatoses, not elsewhere classified |  |
| Q85.9 | Phakomatosis, unspecified |  |
| Q98.0 | Klinefelter syndrome karyotype 47,XXY |  |
| Q98.1 | Klinefelter syndrome, male with more than two X chromosomes |  |
| Q98.2 | Klinefelter syndrome, male with 46,XX karyotype |  |
| Q98.3 | Other male with 46,XX karyotype |  |
| Q98.4 | Klinefelter syndrome, unspecified |  |
| Q99.2 | Fragile X chromosome *(in girls)* |  |
| Q87.0 | Congenital malformation syndromes predominantly affecting facial appearance |  |
| Q87.1 | Congenital malformation syndromes predominantly associated with short stature |  |
| Q87.2 | Congenital malformation syndromes predominantly involving limbs |  |
| Q87.3 | Congenital malformation syndromes involving early overgrowth |  |
| Q87.5 | Other congenital malformation syndromes with other skeletal changes |  |
| Q87.8 | Other specified congenital malformation syndromes, not elsewhere classified |  |

*HES=Hospital Episode Statistics, ICD= International Classification of Diseases version 10, LD=learning disability*

### Trends in coding by type of diagnosis and year of birth

Of young people with LD, 16,026 (31%) had an explicit LD diagnosis, 14,883 (29%) had an underlying “high risk” condition (predominantly Down syndrome), 26,850 (52%) had an underlying “associated” condition (predominantly microcephaly, congenital hydrocephalus, cerebral palsy and congenital anomalies of the brain, Appendix Table 2).

Appendix Table 2 – ICD-10 codes used to indicate young people with learning disability in HES

| Coding cluster | Number | % of children with LD |
| --- | --- | --- |
| Any learning disability | 51,291 |  |
| Co-existing autism | 6,964 | 14% |
| Specific learning disability diagnosis (F7 codes) | 16,026 | 31% |
| Mild LD | 2886 | 6% |
| Moderate LD | 1,265 | 2% |
| Severe or profound LD | 2,659 | 5% |
| Other LD | 11,052 | 22% |
| Hight risk condition | 14,883 | 29% |
| Down syndrome | 7,292 | 14% |
| Other monosomies or trisomies (including Edwards, Patau) | 3,339 | 7% |
| Other congenital anomalies | 3,444 | 7% |
| Congenital anomalies of brain | 465 | 1% |
| Rett syndrome | 454 | 1% |
| Metabolic conditions | 1,044 | 2% |
| Associated conditions | 26,850 | 52% |
| Cerebral palsy | 8,558 | 17% |
| Microcephaly, congenital hydrocephalus, other anomalies of brain | 12,552 | 24% |
| Phakomatoses | 1,570 | 3% |
| Congenital anomalies due to exogenous causes | 1,048 | 2% |
| Klinefelter syndrome | 669 | 1% |
| Other congenital anomalies | 7,497 | 15% |
| Metabolic conditions | 1,437 | 3% |

*HES=Hospital Episode Statistics, ICD= International Classification of Diseases version 10, LD=learning disability*

### Frequency of coding in HES

Diagnoses used to indicate LD or associated conditions were consistently recorded across all calendar years (Appendix Figure 2A). Moderate increase in frequency of recorded diagnoses (across all ages) reflects introduction of financial incentives to improve depth of coding via introduction of PbR.

Appendix Figure 2 – Changes in coding of ICD-10 codes from LD code list over time

*HES=Hospital Episode Statistics, ICD= International Classification of Diseases version 10*

## Code list to identify young people with autism spectrum disorders

### Overview of included codes

There are specific International Classification of Diseases version 10 (ICD-10) codes which can be used to derive a cohort of autistic young people. These are listed in Appendix Table 3.

Appendix Table 3 – ICD-10 codes used to indicate autistic young people in HES

| **ICD-10 code** | **Description** |
| --- | --- |
| F84.0 | Childhood autism |
| F84.1 | Atypical autism |
| F84.3 | Other childhood disintegrative disorder |
| F84.4 | Overactive disorder associated with mental retardation and stereotyped movements |
| F84.5 | Asperger's syndrome |
| F84.8 | Other pervasive developmental disorders |
| F84.9 | Pervasive developmental disorder, unspecified |

*HES=Hospital Episode Statistics, ICD= International Classification of Diseases version 10*

### Trends in coding by calendar year

We plotted the number of recorded diagnoses and new cases for ASD by calendar year and age at admission to explore changes in coding patterns over time. We observed a steady increase in the number of recorded ASD diagnoses and indicated new cases over time (Appendix Figure 3). These results likely reflect improved recognition of ASD and higher coding depth due to the introduction of Payment by Results (PbR), a pay-for-performance system of reimbursing healthcare providers which was introduced in April 2004 (and was fully implemented in 2008/9),^3^ and. We may therefore underestimate the number of young people with ASD who have been admitted to hospital who were born in earlier years, as they would be less likely to have a diagnosis recorded during their hospital admissions.

Appendix Figure 3 – Changes in coding of ICD-10 codes from autism spectrum disorders code list over time

*HES=Hospital Episode Statistics, ICD= International Classification of Diseases version 10*

## Admission type

For each admission, we used the coded admission method recorded in the first episode of care to indicate whether admission was planned, unplanned, or other (as indicated in Appendix Table 4). We excluded admission marked as “other”, which included birth admissions (<300 admissions), and admissions with no admission method (i.e., coded as 99 “not known: a validation error” or 98 “not applicable”, <1500). Among the remaining admissions we indicated pregnancy/maternity-related admissions (based on ICD10, OPCS, and other HES-specific criteria described elsewhere ^4^). Pregnancy/maternity-related admissions were excluded from person time at risk and were not included as events in the analyses (planned or unplanned).

Appendix Table 4 – definition of planned and unplanned admissions^5^

| Value of *admimeth* variable in HES | Meaning | Action |
| --- | --- | --- |
| 11, 12, 13 | Planned admission | Planned |
| 21, 22, 23, 24, 25, 28, 2A, 2B, 2D | Unplanned admission | Unplanned |
| 2C | Unplanned admission for a baby born at home as intended (available from 2013/14) | Other (birth) |
| 31, 32 | Maternity admission | Other (maternity) |
| 82, 83 | Birth of a baby | Other (birth) |
| 81 | Transfer of any admitted patient from other Hospital Provider other than in an Unplanned | Planned |
| 84 | Admission by Admissions Panel of a High Security Psychiatric Hospital, patient not entered on the HSPH Admissions Waiting List (available between 1999 and 2006) | Planned |
| 89 | HSPH Admissions Waiting List of a High Security Psychiatric Hospital (available between 1999 and 2006) | Planned |
| 98 | Not applicable (available from 1996/97) | Other |
| 99 | Not known: a validation error | Other |

## Main reasons for hospital admissions

We indicated 10 most commonly recorded diagnoses (based on 3-letter ICD-10 codes) at ages 10-15 years, 16-18 years and 19-24 years. Using this information, we generated broader groups of common reasons for hospital admissions.

Appendix Table 5 – 10 most common 3-letter ICD-10 codes recorded as primary diagnosis by age for planned admissions for young people with learning disabilities

| 10-15 years old | | | | 16-18 years old | | | | 19-24 years old | | | |
| --- | --- | --- | --- | --- | --- | --- | --- | --- | --- | --- | --- |
| ICD-10 | **Description** | **Count** | **%** | **ICD-10** | **Description** | **Count** | **%** | **ICD-10** | **Description** | **Count** | **%** |
| Z75 | Problems related to medical facilities and other health care | 46592 | 24% | Z75 | Problems related to medical facilities and other health care | 13740 | 20% | N18 | Chronic kidney disease | 7799 | 14% |
| G80 | Cerebral palsy | 9983 | 5% | N18 | Chronic kidney disease | 4956 | 7% | Z75 | Problems related to medical facilities and other health care | 6931 | 12% |
| F79 | Unspecified mental retardation | 8145 | 4% | G80 | Cerebral palsy | 3639 | 5% | R69 | Unknown and unspecified causes of morbidity | 3824 | 7% |
| R69 | Unknown and unspecified causes of morbidity | 6770 | 4% | R69 | Unknown and unspecified causes of morbidity | 3102 | 5% | K02 | Dental caries | 2202 | 4% |
| N18 | Chronic kidney disease | 6657 | 3% | K02 | Dental caries | 2028 | 3% | G80 | Cerebral palsy | 2035 | 4% |
| K02 | Dental caries | 5546 | 3% | G40 | Epilepsy | 1500 | 2% | G40 | Epilepsy | 1100 | 2% |
| E76 | Disorders of glycosaminoglycan metabolism | 5345 | 3% | F72 | Severe mental retardation | 1420 | 2% | Z45 | Adjustment and management of implanted device | 784 | 1% |
| F72 | Severe mental retardation | 5115 | 3% | M41 | Scoliosis | 1207 | 2% | Z43 | Attention to artificial openings | 662 | 1% |
| G40 | Epilepsy | 4724 | 2% | E76 | Disorders of glycosaminoglycan metabolism | 859 | 1% | K50 | Crohn disease [regional enteritis] | 610 | 1% |
| M41 | Scoliosis | 3213 | 2% | F79 | Unspecified mental retardation | 782 | 1% | F72 | Severe mental retardation | 538 | 1% |

Appendix Table 6 – 10 most common 3-letter ICD-10 codes recorded as primary diagnosis by age for planned admissions for young people with autism spectrum disorders

| 10-15 years old | | | | 16-18 years old | | | | 19-24 years old | | | |
| --- | --- | --- | --- | --- | --- | --- | --- | --- | --- | --- | --- |
| ICD-10 | **Description** | **Count** | **%** | **ICD-10** | **Description** | **Count** | **%** | **ICD-10** | **Description** | **Count** | **%** |
| Z75 | Problems related to medical facilities and other health care | 6007 | 13% | K02 | Dental caries | 1641 | 8% | K02 | Dental caries | 1854 | Z75 |
| K02 | Dental caries | 4740 | 10% | N18 | Chronic kidney disease | 1268 | 6% | N18 | Chronic kidney disease | 1611 | K02 |
| F84 | Pervasive developmental disorders | 1772 | 4% | Z75 | Problems related to medical facilities and other health care | 949 | 4% | K50 | Crohn disease [regional enteritis] | 1085 | F84 |
| R69 | Unknown and unspecified causes of morbidity | 1530 | 3% | K50 | Crohn disease [regional enteritis] | 795 | 4% | R69 | Unknown and unspecified causes of morbidity | 831 | R69 |
| G40 | Epilepsy | 1028 | 2% | R69 | Unknown and unspecified causes of morbidity | 704 | 3% | C81 | Hodgkin lymphoma | 460 | G40 |
| C91 | Lymphoid leukaemia | 977 | 2% | L60 | Nail disorders | 510 | 2% | K51 | Ulcerative colitis | 454 | C91 |
| L60 | Nail disorders | 904 | 2% | F84 | Pervasive developmental disorders | 468 | 2% | K01 | Embedded and impacted teeth | 343 | L60 |
| K07 | Dentofacial anomalies [including malocclusion] | 850 | 2% | C91 | Lymphoid leukaemia | 450 | 2% | Z75 | Problems related to medical facilities and other health care | 342 | K07 |
| K50 | Crohn disease [regional enteritis] | 817 | 2% | G40 | Epilepsy | 369 | 2% | E84 | Cystic fibrosis | 321 | K50 |
| K01 | Embedded and impacted teeth | 614 | 1% | K07 | Dentofacial anomalies [including malocclusion] | 362 | 2% | R10 | Abdominal and pelvic pain | 310 | K01 |

Appendix Table 7 – Code list for main reason for planned admissions

| Primary diagnoses group | | Definition |
| --- | --- | --- |
| Respite care | | ICD-10: Z75.5 |
| Cerebral palsy and other paralytic syndromes | | ICD-10: G80- G83 |
| Epilepsy | | ICD-10: F80.3, G40.0, G40.1, G40.2, G40.3, G40.4, G40.6, G40.7, G41, G40.8, G40.9, R56.8, Y46.0, Y46.1, Y46.2, Y46.3, Y46.4, Y46.5, Y46.6 |
| Specific learning disability or autism spectrum disorder diagnosis | | ICD-10: F70-F79, F84 |
| Dental | Dental Caries | ICD-10: K02 |
|  | Tooth extraction | OPCS: F09-F10 |
| Kidney Disease | | ICD-10: N17, N18, N19 |
| Inflammatory bowel disease | | ICD-10: K50, K51, K52 |
| Unknown and unspecified | | R69 |
| Surgical | | if there was at least one recorded OPCS procedure code, excluding:   - obstetric scans, other physiological assessments and ultrasound (R36-R43) - diagnostic imaging, testing and rehabilitation (codes starting with U) - other non-operations (Y90) - miscellaneous operations (X28-X68) including infusion of therapeutic substance, injection, blood transfusion, oxygen, administration of vaccine - Specified drug therapy (X70-X97) |

Appendix Table 8 – 10 most common 3-letter ICD-10 codes recorded as primary diagnosis by age for unplanned admissions for young people with learning disabilities

| 10-15 years old | | | | 16-18 years old | | | | 19-24 years old | | | |
| --- | --- | --- | --- | --- | --- | --- | --- | --- | --- | --- | --- |
| ICD-10 | **Description** | **Count** | **%** | **ICD-10** | **Description** | **Count** | **%** | **ICD-10** | **Description** | **Count** | **%** |
| G40 | Epilepsy | 8907 | 12% | G40 | Epilepsy | 3622 | 11% | G40 | Epilepsy | 3607 | 9% |
| J22 | Unspecified acute lower respiratory infection | 4647 | 6% | J22 | Unspecified acute lower respiratory infection | 2095 | 6% | R10 | Abdominal and pelvic pain | 2148 | 5% |
| R56 | Convulsions, not elsewhere classified | 2744 | 4% | R10 | Abdominal and pelvic pain | 1377 | 4% | J18 | Pneumonia, organism unspecified | 1415 | 3% |
| J18 | Pneumonia, organism unspecified | 2157 | 3% | J18 | Pneumonia, organism unspecified | 1148 | 3% | E10 | Type 1 diabetes mellitus | 1402 | 3% |
| R10 | Abdominal and pelvic pain | 2048 | 3% | R56 | Convulsions, not elsewhere classified | 1008 | 3% | J22 | Unspecified acute lower respiratory infection | 1285 | 3% |
| T85 | Complications of other internal prosthetic devices, implants and grafts | 2034 | 3% | T39 | Poisoning by nonopioid analgesics, antipyretics and antirheumatics | 972 | 3% | T39 | Poisoning by nonopioid analgesics, antipyretics and antirheumatics | 1276 | 3% |
| R11 | Nausea and vomiting | 1902 | 3% | T85 | Complications of other internal prosthetic devices, implants and grafts | 921 | 3% | J69 | Pneumonitis due to solids and liquids | 1186 | 3% |
| R51 | Headache | 1746 | 2% | R51 | Headache | 819 | 2% | N39 | Other disorders of urinary system | 1078 | 3% |
| Z75 | Problems related to medical facilities and other health care | 1654 | 2% | E10 | Type 1 diabetes mellitus | 807 | 2% | R56 | Convulsions, not elsewhere classified | 1012 | 2% |
| R69 | Unknown and unspecified causes of morbidity | 1641 | 2% | R11 | Nausea and vomiting | 723 | 2% | R51 | Headache | 995 | 2% |

Appendix Table 9 – 10 most common 3-letter ICD-10 codes recorded as primary diagnosis by age for unplanned admissions for young people with autism spectrum disorders

| 10-15 years old | | | | 16-18 years old | | | | 19-24 years old | | | |
| --- | --- | --- | --- | --- | --- | --- | --- | --- | --- | --- | --- |
| ICD-10 | **Description** | **Count** | **%** | **ICD-10** | **Description** | **Count** | **%** | **ICD-10** | **Description** | **Count** | **%** |
| R10 | Abdominal and pelvic pain | 2353 | 6% | T39 | Poisoning by nonopioid analgesics, antipyretics and antirheumatics | 2575 | 11% | T39 | Poisoning by nonopioid analgesics, antipyretics and antirheumatics | 2315 | 8% |
| G40 | Epilepsy | 2071 | 6% | R10 | Abdominal and pelvic pain | 1523 | 6% | R10 | Abdominal and pelvic pain | 1932 | 7% |
| T39 | Poisoning by nonopioid analgesics, antipyretics and antirheumatics | 1844 | 5% | T43 | Poisoning by psychotropic drugs, not elsewhere classified | 1113 | 5% | T43 | Poisoning by psychotropic drugs, not elsewhere classified | 1155 | 4% |
| R56 | Convulsions, not elsewhere classified | 1473 | 4% | G40 | Epilepsy | 914 | 4% | G40 | Epilepsy | 981 | 3% |
| R45 | Symptoms and signs involving emotional state | 1098 | 3% | R45 | Symptoms and signs involving emotional state | 827 | 3% | F60 | Specific personality disorders | 842 | 3% |
| J45 | Asthma | 976 | 3% | R56 | Convulsions, not elsewhere classified | 669 | 3% | E10 | Type 1 diabetes mellitus | 674 | 2% |
| E10 | Type 1 diabetes mellitus | 912 | 2% | F84 | Pervasive developmental disorders | 652 | 3% | R07 | Pain in throat and chest | 658 | 2% |
| K59 | Other functional intestinal disorders | 861 | 2% | E10 | Type 1 diabetes mellitus | 558 | 2% | R45 | Symptoms and signs involving emotional state | 592 | 2% |
| S52 | Fracture of forearm | 839 | 2% | J45 | Asthma | 374 | 2% | R56 | Convulsions, not elsewhere classified | 592 | 2% |
| T43 | Poisoning by psychotropic drugs, not elsewhere classified | 749 | 2% | F32 | Depressive episode | 360 | 1% | F84 | Pervasive developmental disorders | 581 | 2% |

Appendix Table 10 – Code list for main reason for unplanned admissions

| Primary diagnoses group | | ICD-10 codes |
| --- | --- | --- |
| Epilepsy | | F80.3, G40.0, G40.1, G40.2, G40.3, G40.4, G40.6, G40.7, G40.8, G40.9, G41, R56.8, Y46.0, Y46.1, Y46.2, Y46.3, Y46.4, Y46.5, Y46.6 |
| Respiratory infections | | A15-A19, A48.1, A48.2, B59, J00-J06, J10-J22, J32, J36, J37, J39.0, J39.1, J40-J42, J43.0, J44.0, J47, J56, J85, J86, J98.8, N74.0, N74.1 |
| Other infections (enteric / gastrointestinal / genitourinary) | | A00-A09, I88.0, K23.0, K23.1, K25-K28, K29.3, K29.4, K29.5, K35-K37, K52.8, K52.9, K61, K63.0, K63.2, K65.0, K67.8, K90.8, K93.0, N30.0, N34.1, N35.1, N37, N39.0, N41.0, N41.1, N41.2, N41.3, N43.1, N45, N48.1, N48.2, N49, N51, N70-N74, N75.1, N76.4, N87, R11 |
| /Symptoms and signs | Abdominal pain | R10-R19, K59 |
|  | General symptoms and signs | R50-R69 (excluding R56.8) |
|  | Respiratory and circulatory symptoms | R00-R09 |
|  | Other symptoms | R20-49, R70-R99 |
| Injury | Injury, poisoning and certain other consequences of external causes | S00-T98 |
|  | Injury due to self-harm | As above, and additionally any secondary diagnosis included: X60-X84, Z91.5 |
| Mental Health Problems | Mental and behavioural disorders due to psychoactive substance use | F10-F19 |
|  | Schizophrenia, schizotypal and delusional disorders | F20-F29 |
|  | Mood [affective] disorders | F30-F39 |
|  | Adult personality disorders | F60, F61, F69 |

# Additional results

## Secondary outcomes: planned hospital admissions

Appendix Figure 4 – Crude surgical and non-surgical planned admission rates for young people with learning disabilities (LD) and autism spectrum disorders (ASD)

Appendix Table 11 – changes in length of stay for planned admissions for young people with learning disabilities

| **Age at admission** | **Mean (SD)** | **Median (IQR)** | **Day cases N (%)** | **Overnight stay N (%)** | **2-6 days N (%)** | **>7 days N (%)** |
| --- | --- | --- | --- | --- | --- | --- |
| 10 | 1.9 (10.0) | 1 (0, 2) | 13,545 (41%) | 9,092 (28%) | 8,772 (27%) | 1,277 (4%) |
| 11 | 1.9 (10.3) | 1 (0, 2) | 13,525 (42%) | 8,636 (27%) | 8,890 (27%) | 1,300 (4%) |
| 12 | 2.0 (11.9) | 1 (0, 2) | 13,522 (42%) | 8,369 (26%) | 8,803 (27%) | 1,361 (4%) |
| 13 | 2.3 (16.7) | 1 (0, 2) | 13,250 (43%) | 7,767 (25%) | 8,388 (27%) | 1,434 (5%) |
| 14 | 2.7 (33.4) | 1 (0, 2) | 14,529 (46%) | 7,441 (24%) | 8,151 (26%) | 1,520 (5%) |
| 15 | 2.3 (22.4) | 1 (0, 2) | 15,594 (49%) | 6,937 (22%) | 7,569 (24%) | 1,470 (5%) |
| 16 | 2.8 (37.5) | 0 (0, 2) | 14,248 (51%) | 5,518 (20%) | 6,813 (24%) | 1,287 (5%) |
| 17 | 3.5 (43.4) | 0 (0, 2) | 12,328 (53%) | 4,279 (18%) | 5,651 (24%) | 1,124 (5%) |
| 18 | 4.6 (47.5) | 0 (0, 2) | 9,811 (57%) | 2,542 (15%) | 3,764 (22%) | 1,038 (6%) |
| 19 | 4.9 (59.6) | 0 (0, 2) | 8,586 (63%) | 1,685 (12%) | 2,547 (19%) | 891 (6%) |
| 20 | 5.4 (62.2) | 0 (0, 1) | 7,820 (65%) | 1,276 (11%) | 2,132 (18%) | 813 (7%) |
| 21 | 4.5 (45.2) | 0 (0, 1) | 6,867 (66%) | 963 (9%) | 1,785 (17%) | 730 (7%) |
| 22 | 4.5 (47.7) | 0 (0, 1) | 5,446 (67%) | 700 (9%) | 1,406 (17%) | 593 (7%) |
| 23 | 3.3 (34.2) | 0 (0, 1) | 4,204 (66%) | 551 (9%) | 1,116 (18%) | 462 (7%) |
| 24 | 2.9 (22.6) | 0 (0, 1) | 3,422 (66%) | 463 (9%) | 909 (18%) | 355 (7%) |

*IQR: interquartile range; SD: standard deviation*

Appendix Table 12 – changes in length of stay for planned admissions for young people with autism spectrum disorders

| **Age at admission** | **Mean (SD)** | **Median (IQR)** | **Day cases N (%)** | **Overnight stay N (%)** | **2-6 days N (%)** | **>7 days N (%)** |
| --- | --- | --- | --- | --- | --- | --- |
| 10 | 1.7 (13.8) | 0 (0, 1) | 4,384 (60%) | 1,842 (25%) | 883 (12%) | 198 (3%) |
| 11 | 2.6 (22.4) | 0 (0, 1) | 4,707 (63%) | 1,568 (21%) | 946 (13%) | 231 (3%) |
| 12 | 3.2 (33.7) | 0 (0, 1) | 5,312 (67%) | 1,448 (18%) | 847 (11%) | 283 (4%) |
| 13 | 3.5 (34.6) | 0 (0, 1) | 5,437 (69%) | 1,285 (16%) | 881 (11%) | 320 (4%) |
| 14 | 3.7 (29.5) | 0 (0, 1) | 5,446 (68%) | 1,324 (17%) | 841 (11%) | 359 (5%) |
| 15 | 6.2 (48.0) | 0 (0, 1) | 5,713 (69%) | 1,235 (15%) | 914 (11%) | 477 (6%) |
| 16 | 5.3 (46.2) | 0 (0, 1) | 5,875 (72%) | 943 (12%) | 878 (11%) | 478 (6%) |
| 17 | 5.2 (47.5) | 0 (0, 1) | 5,541 (74%) | 769 (10%) | 762 (10%) | 449 (6%) |
| 18 | 4.9 (52.2) | 0 (0, 0) | 4,531 (76%) | 626 (11%) | 475 (8%) | 298 (5%) |
| 19 | 5.9 (58.2) | 0 (0, 0) | 3,908 (76%) | 561 (11%) | 386 (8%) | 268 (5%) |
| 20 | 8.5 (80.3) | 0 (0, 0) | 3,333 (77%) | 403 (9%) | 279 (6%) | 290 (7%) |
| 21 | 5.2 (52.3) | 0 (0, 0) | 3,133 (80%) | 322 (8%) | 262 (7%) | 207 (5%) |
| 22 | 4.5 (52.6) | 0 (0, 0) | 3,000 (83%) | 236 (7%) | 220 (6%) | 165 (5%) |
| 23 | 3.0 (34.8) | 0 (0, 0) | 2,394 (83%) | 210 (7%) | 158 (6%) | 107 (4%) |
| 24 | 4.2 (36.2) | 0 (0, 0) | 1,889 (84%) | 157 (7%) | 104 (5%) | 99 (4%) |

*IQR: interquartile range; SD: standard deviation*

## Secondary outcomes: unplanned hospital admissions

Appendix Table 13 – Changes in length of stay for unplanned admissions for young people with learning disabilities

| **Age at admission** | **Mean (SD)** | **Median (IQR)** | **Day cases N (%)** | **Overnight stay N (%)** | **2-6 days N (%)** | **>7 days N (%)** |
| --- | --- | --- | --- | --- | --- | --- |
| 10 | 3.3 (13.8) | 1 (0, 3) | 3,846 (33%) | 3,156 (27%) | 3,448 (29%) | 1,288 (11%) |
| 11 | 3.6 (14.7) | 1 (0, 3) | 3,792 (32%) | 3,178 (27%) | 3,428 (29%) | 1,413 (12%) |
| 12 | 3.6 (14.4) | 1 (0, 3) | 4,016 (34%) | 3,073 (26%) | 3,368 (28%) | 1,409 (12%) |
| 13 | 4.4 (26.6) | 1 (0, 3) | 3,804 (33%) | 2,958 (25%) | 3,337 (29%) | 1,556 (13%) |
| 14 | 4.9 (30.2) | 1 (0, 3) | 4,055 (33%) | 2,971 (24%) | 3,535 (29%) | 1,664 (14%) |
| 15 | 5.0 (25.8) | 1 (0, 3) | 4,130 (33%) | 3,056 (25%) | 3,480 (28%) | 1,687 (14%) |
| 16 | 6.1 (32.8) | 1 (0, 4) | 4,017 (33%) | 2,910 (24%) | 3,456 (28%) | 1,887 (15%) |
| 17 | 7.5 (47.2) | 1 (0, 4) | 3,574 (31%) | 2,663 (23%) | 3,306 (29%) | 1,921 (17%) |
| 18 | 10.0 (57.5) | 1 (0, 5) | 2,974 (28%) | 2,306 (22%) | 3,190 (30%) | 2,028 (19%) |
| 19 | 11.9 (81.9) | 2 (0, 5) | 2,585 (28%) | 1,999 (21%) | 2,903 (31%) | 1,851 (20%) |
| 20 | 10.0 (58.6) | 2 (0, 5) | 2,252 (27%) | 1,799 (22%) | 2,539 (31%) | 1,610 (20%) |
| 21 | 9.1 (52.8) | 2 (0, 5) | 2,023 (28%) | 1,614 (22%) | 2,260 (31%) | 1,417 (19%) |
| 22 | 8.4 (44.8) | 1 (0, 5) | 1,852 (29%) | 1,391 (21%) | 1,970 (30%) | 1,260 (19%) |
| 23 | 7.7 (34.3) | 1 (0, 5) | 1,573 (28%) | 1,213 (22%) | 1,731 (31%) | 1,053 (19%) |
| 24 | 7.8 (34.3) | 1 (0, 5) | 1,255 (29%) | 968 (22%) | 1,338 (31%) | 805 (18%) |

*IQR: interquartile range; SD: standard deviation*

Appendix Table 14 – Changes in length of stay for unplanned admissions for young people with autism spectrum disorders

| **Age at admission** | **Mean (SD)** | **Median (IQR)** | **Day cases N (%)** | **Overnight stay N (%)** | **2-6 days N (%)** | **>7 days N (%)** |
| --- | --- | --- | --- | --- | --- | --- |
| 10 | 2.1 (17.8) | 1 (0, 2) | 1,744 (40%) | 1,445 (33%) | 1,013 (23%) | 203 (5%) |
| 11 | 2.4 (13.7) | 1 (0, 2) | 1,787 (38%) | 1,469 (32%) | 1,150 (25%) | 244 (5%) |
| 12 | 3.3 (20.4) | 1 (0, 2) | 2,031 (38%) | 1,671 (31%) | 1,302 (24%) | 326 (6%) |
| 13 | 4.2 (24.3) | 1 (0, 2) | 2,179 (37%) | 1,896 (32%) | 1,423 (24%) | 441 (7%) |
| 14 | 5.4 (31.5) | 1 (0, 2) | 2,758 (37%) | 2,329 (31%) | 1,756 (24%) | 611 (8%) |
| 15 | 6.0 (30.8) | 1 (0, 2) | 3,281 (37%) | 2,723 (30%) | 2,120 (24%) | 860 (10%) |
| 16 | 6.4 (32.9) | 1 (0, 2) | 3,078 (36%) | 2,440 (29%) | 1,993 (24%) | 930 (11%) |
| 17 | 6.0 (35.8) | 1 (0, 2) | 3,005 (38%) | 2,124 (27%) | 1,926 (24%) | 919 (12%) |
| 18 | 8.2 (49.9) | 1 (0, 3) | 2,749 (36%) | 2,000 (26%) | 1,853 (24%) | 1,075 (14%) |
| 19 | 8.9 (56.8) | 1 (0, 3) | 2,447 (36%) | 1,677 (25%) | 1,686 (25%) | 996 (15%) |
| 20 | 7.5 (35.8) | 1 (0, 3) | 2,148 (36%) | 1,507 (25%) | 1,492 (25%) | 869 (14%) |
| 21 | 9.6 (49.2) | 1 (0, 3) | 1,794 (34%) | 1,298 (25%) | 1,358 (26%) | 823 (16%) |
| 22 | 7.6 (37.2) | 1 (0, 3) | 1,479 (35%) | 1,027 (24%) | 1,085 (26%) | 603 (14%) |
| 23 | 7.2 (36.6) | 1 (0, 3) | 1,181 (35%) | 808 (24%) | 888 (26%) | 524 (15%) |
| 24 | 8.2 (35.4) | 1 (0, 4) | 864 (34%) | 645 (25%) | 649 (25%) | 399 (16%) |

*IQR: interquartile range; SD: standard deviation*

## Sensitivity analyses

### Mortality rates

Overall, 2,327 (4.7%) of young people with LD and 292 (0.6%) autistic young people died during the study period. Mortality rates were 4.0 and 0.5 deaths per 1000 person-years, respectively (Appendix Figure 5). Results from sensitivity analyses excluding children who died were consistent with results from the main analysis (Appendix Table 15).

Appendix Figure 5 – Crude mortality rates for young people with learning disabilities and autism spectrum disorders compared to the general population of young people aged 10-24 years old in England and Wales^6,7^

Appendix Table 15 – Adjusted hospital admission rates and rate ratios (95% confidence intervals)) for hospital admissions for young people with learning disabilities (LD) and autism spectrum disorders (ASD), excluding young people who died during the study period

|  | **Young people with LD** | | **Autistic young people** | |
| --- | --- | --- | --- | --- |
|  | **Planned admissions** | **Unplanned admissions** | **Planned admissions** | **Unplanned admissions** |
| **Admission rates at baseline for each age group per 100 person-years (intercept)** | | | |  |
| Age 10 | 6.8 (6.5, 7.2) | 2.5 (2.4, 2.6) | 5.4 (5.1, 5.6) | 4.1 (3.9, 4.2) |
| Age 16 | 7.0 (6.6, 7.5) | 2.8 (2.6, 3.0) | 7.0 (6.6, 7.4) | 7.9 (7.4, 8.4) |
| Age 19 | 3.9 (3.7, 4.1) | 2.9 (2.7, 3.1) | 5.6 (5.3, 5.9) | 8.4 (8.0, 8.8) |
| **All estimates below are rate ratios:** | | | |  |
| **Change in admission rate per year by age group (slope)** | | | |  |
| Age 10-15 | 0.99 (0.98, 0.99) | 1.03 (1.02, 1.03) | 1.06 (1.05, 1.06) | 1.16 (1.15, 1.17) |
| Age 16-18 | 0.86 (0.85, 0.88) | 1.01 (0.99, 1.03) | 0.95 (0.93, 0.97) | 1.01 (0.99, 1.03) |
| Aged 19-24 | 1.00 (0.99, 1.00) | 1.03 (1.02, 1.04) | 1.05 (1.04, 1.07) | 1.03 (1.02, 1.04) |
| **Sex** |  |  |  |  |
| Male | 1.00 | 1.00 | 1.00 | 1.00 |
| Female | 0.89 (0.86, 0.91) | 1.04 (1.01, 1.07) | 0.93 (0.91, 0.96) | 1.57 (1.52, 1.61) |
| **IMD Quintile** | |  |  |  |
| Q1: Most deprived 20% | 0.81 (0.78, 0.85) | 1.25 (1.19, 1.31) | 0.93 (0.90, 0.97) | 1.03 (0.99, 1.07) |
| Q2 | 0.88 (0.84, 0.92) | 1.15 (1.09, 1.21) | 0.93 (0.89, 0.97) | 1.00 (0.96, 1.04) |
| Q3 | 0.95 (0.91, 1.00) | 1.08 (1.03, 1.14) | 0.95 (0.91, 0.99) | 1.03 (0.99, 1.07) |
| Q4 | 0.99 (0.94, 1.03) | 1.05 (0.99, 1.11) | 0.97 (0.93, 1.02) | 0.99 (0.94, 1.03) |
| Q5: Least deprived 20% | 1 | 1 | 1 | 1 |
| **Year of birth** | |  |  |  |
| 1990-1993 | 1.52 (1.47, 1.57) | 1.43 (1.38, 1.49) | 0.90 (0.87, 0.93) | 0.76 (0.73, 0.79) |
| 1994-1997 | 1.22 (1.18, 1.26) | 1.20 (1.16, 1.25) | 0.87 (0.85, 0.90) | 0.78 (0.75, 0.80) |
| 1998-2001 | 1 | 1 | 1 | 1 |
| **Presence of different groups of chronic conditions (out of 7)** | | |  |  |
| None | 1 | 1 | 1 | 1 |
| 1 | 1.90 (1.83, 1.98) | 1.63 (1.56, 1.71) | 1.56 (1.51, 1.60) | 1.53 (1.49, 1.58) |
| 2 | 3.27 (3.14, 3.41) | 2.63 (2.51, 2.75) | 2.66 (2.56, 2.77) | 2.27 (2.18, 2.35) |
| 3+ | 8.57 (8.24, 8.90) | 6.60 (6.33, 6.89) | 5.93 (5.66, 6.22) | 4.26 (4.07, 4.46) |

*IMD=Index of Multiple Deprivation.* *All estimates are mutually adjusted for covariates listed in the table.*

### Analyses stratified by year of birth

Appendix Table 16 – Adjusted hospital admission rates and rate ratios (95% confidence intervals) for planned admissions for young people with learning disabilities by year of birth category derived from multilevel negative binomial models

|  | **Young people born in 1990-1993** | **Young people born in 1994-1997** | | **Young people born in 1998-2001*** |
| --- | --- | --- | --- | --- |
| **Admission rates at baseline for each age group per 100 person-years (intercept)** | | | | |
| Age 10 | 12.1 (11.1, 13.1) | 7.7 (7.1, 8.4) | | 5.9 (5.4, 6.4) |
| Age 16 | 11.3 (10.2, 12.5) | 8.8 (8.0, 9.7) | | 6.1 (5.6, 6.8) |
| Age 19 | 6.7 (6.1, 7.3) | 5.2 (4.8, 5.7) | | 4.4 (3.7, 5.2) |
| **Change in admission rate per year by age group (slope)** | | | |  |
| Age 10-15 | 0.97 (0.96, 0.98) | 1.00 (0.99, 1.00) | | 0.99 (0.99, 1.00) |
| Age 16-18 | 0.90 (0.87, 0.93) | 0.85 (0.83, 0.88) | | 0.83 (0.81, 0.86) |
| Aged 19-24 | 1.01 (1.00, 1.02) | 0.94 (0.93, 0.96) | | 0.69 (0.62, 0.77) |
| **Sex** |  |  | |  |
| Male | 1.0 | 1.0 | | 1.0 |
| Female | 0.85 (0.81, 0.89) | 0.88 (0.84, 0.93) | | 0.93 (0.89, 0.98) |
| **IMD Quintile** | |  | |  |
| Q1: Most deprived 20% | 0.79 (0.73, 0.85) | 0.85 (0.79, 0.91) | | 0.79 (0.74, 0.85) |
| Q2 | 0.84 (0.77, 0.91) | 0.92 (0.85, 0.99) | | 0.88 (0.82, 0.95) |
| Q3 | 0.95 (0.87, 1.03) | 0.98 (0.91, 1.06) | | 0.91 (0.84, 0.98) |
| Q4 | 1.00 (0.92, 1.09) | 0.98 (0.90, 1.06) | | 0.96 (0.89, 1.04) |
| Q5: Least deprived 20% | 1.0 | 1.0 | | 1.0 |
| **Presence of different groups of chronic conditions (out of 7)** | | |  |  |
| None | 1.0 | 1.0 | | 1.0 |
| 1 | 1.79 (1.68, 1.92) | 1.94 (1.81, 2.07) | 2.07 (1.93, 2.23) | |
| 2 | 3.01 (2.80, 3.24) | 3.36 (3.13, 3.60) | 3.72 (3.45, 4.00) | |
| 3+ | 7.59 (7.10, 8.13) | 9.36 (8.77, 9.98) | 10.96 (10.25, 11.71) | |

**note that follow-up was available up to max of age 20 years old for the oldest children in the cohort (born in 1998)*

Appendix Table 17 – Adjusted hospital admission rates and rate ratios (95% confidence intervals) for planned admissions for autistic young people by year of birth category derived from multilevel negative binomial models

|  | **Young people born in 1990-1993** | **Young people born in 1994-1997** | | **Young people born in 1998-2001*** |
| --- | --- | --- | --- | --- |
| **Admission rates at baseline for each age group per 100 person-years (intercept)** | | | |  |
| Age 10 | 5.3 (4.9, 5.8) | 4.8 (4.5, 5.1) | | 4.9 (4.6, 5.2) |
| Age 16 | 5.4 (4.7, 6.2) | 5.7 (5.2, 6.3) | | 7.8 (7.1, 8.6) |
| Age 19 | 5.1 (4.7, 5.7) | 5.5 (5.0, 6.0) | | 6.9 (5.7, 8.4) |
| **Change in admission rate per year by age group (slope)** | | | |  |
| Age 10-15 | 1.02 (1.00, 1.04) | 1.05 (1.03, 1.06) | | 1.08 (1.07, 1.10) |
| Age 16-18 | 1.00 (0.95, 1.06) | 0.97 (0.94, 1.01) | | 0.90 (0.87, 0.94) |
| Aged 19-24 | 1.07 (1.05, 1.09) | 1.00 (0.98, 1.02) | | 0.76 (0.67, 0.87) |
| **Sex** |  |  | |  |
| Male | 1 | 1 | | 1 |
| Female | 0.92 (0.86, 0.98) | 0.90 (0.85, 0.94) | | 0.96 (0.92, 1.01) |
| **IMD Quintile** | |  | |  |
| Q1: Most deprived 20% | 0.91 (0.84, 0.99) | 0.00 (0.84, 0.97) | | 0.97 (0.91, 1.04) |
| Q2 | 0.94 (0.86, 1.02) | 0.95 (0.89, 1.02) | | 0.91 (0.85, 0.98) |
| Q3 | 0.98 (0.90, 1.07) | 0.96 (0.89, 1.03) | | 0.93 (0.86, 1.00) |
| Q4 | 0.99 (0.90, 1.08) | 0.96 (0.89, 1.03) | | 0.98 (0.90, 1.05) |
| Q5: Least deprived 20% | 1 | 1 | | 1 |
| **Presence of different groups of chronic conditions (out of 7)** | | |  |  |
| None | 1 | 1 | | 1 |
| 1 | 1.57 (1.47, 1.67) | 1.58 (1.50, 1.66) | | 1.54 (1.47, 1.62) |
| 2 | 2.51 (2.31, 2.72) | 2.94 (2.76, 3.13) | | 2.59 (2.43, 2.76) |
| 3+ | 5.73 (5.20, 6.32) | 6.48 (6.00, 6.99) | | 6.40 (5.93, 6.90) |

**note that follow-up was available up to max of age 20 years old for the oldest children in the cohort (born in 1998)*

Appendix Table 18 – Adjusted hospital admission rates and rate ratios (95% confidence intervals) for unplanned admissions for young people with learning disabilities by year of birth category derived from multilevel negative binomial models

|  | **Young people born in 1990-1993** | **Young people born in 1994-1997** | | **Young people born in 1998-2001*** |
| --- | --- | --- | --- | --- |
| **Admission rates at baseline for each age group per 100 person-years (intercept)** | | | | |
| Age 10 | 3.8 (3.5, 4.2) | 2.8 (2.6, 3.1) | | 2.2 (1.9, 2.4) |
| Age 16 | 4.2 (3.7, 4.7) | 3.2 (2.9, 3.6) | | 2.6 (2.3, 2.9) |
| Age 19 | 4.5 (4.1, 4.9) | 3.8 (3.5, 4.2) | | 3.0 (2.5, 3.6) |
| **Change in admission rate per year by age group (slope)** | | | | |
| Age 10-15 | 1.02 (1.01, 1.03) | 1.03 (1.02, 1.04) | | 1.03 (1.02, 1.04) |
| Age 16-18 | 1.02 (0.99, 1.06) | 1.03 (1.00, 1.07) | | 0.98 (0.95, 1.01) |
| Aged 19-24 | 1.04 (1.03, 1.05) | 0.98 (0.97, 1.00) | | 0.79 (0.70, 0.88) |
| **Sex** |  |  | |  |
| Male | 1 | 1 | | 1 |
| Female | 0.93 (0.88, 0.98) | 1.05 (1.00, 1.11) | | 1.11 (1.05, 1.17) |
| **IMD Quintile** | |  | |  |
| Q1: Most deprived 20% | 1.30 (1.20, 1.41) | 1.27 (1.17, 1.37) | | 1.19 (1.09, 1.30) |
| Q2 | 1.11 (1.01, 1.21) | 1.17 (1.07, 1.27) | | 1.16 (1.06, 1.27) |
| Q3 | 1.08 (0.98, 1.18) | 1.04 (0.96, 1.14) | | 1.12 (1.01, 1.23) |
| Q4 | 1.05 (0.96, 1.15) | 1.06 (0.97, 1.16) | | 1.01 (0.91, 1.11) |
| Q5: Least deprived 20% | 1 | 1 | | 1 |
| **Presence of different groups of chronic conditions (out of 7)** | | |  |  |
| None | 1 | 1 | | 1 |
| 1 | 1.59 (1.48, 1.70) | 1.75 (1.62, 1.88) | | 1.65 (1.51, 1.80) |
| 2 | 2.98 (2.77, 3.22) | 2.80 (2.59, 3.03) | | 2.62 (2.39, 2.86) |
| 3+ | 8.60 (8.01, 9.23) | 8.54 (7.96, 9.17) | | 8.32 (7.69, 9.01) |

**note that follow-up was available up to max of age 20 years old for the oldest children in the cohort (born in 1998)*

Appendix Table 19 – Adjusted hospital admission rates and rate ratios (95% confidence intervals) for unplanned admissions for autistic young people by year of birth category derived from multilevel negative binomial models

|  | **Young people born in 1990-1993** | **Young people born in 1994-1997** | | **Young people born in 1998-2001*** |
| --- | --- | --- | --- | --- |
| **Admission rates at baseline for each age group per 100 person-years (intercept)** | | | | |
| Age 10 | 3.1 (2.9, 3.4) | 3.3 (3.1, 3.5) | | 3.9 (3.7, 4.2) |
| Age 16 | 4.9 (4.3, 5.5) | 5.9 (5.4, 6.5) | | 9.0 (8.3, 9.8) |
| Age 19 | 6.5 (6.0, 7.1) | 7.2 (6.7, 7.8) | | 12.8 (10.9, 15.1) |
| **Change in admission rate per year by age group (slope)** | | | | |
| Age 10-15 | 1.12 (1.10, 1.14) | 1.14 (1.12, 1.15) | | 1.19 (1.18, 1.20) |
| Age 16-18 | 1.08 (1.03, 1.14) | 1.03 (0.99, 1.07) | | 0.97 (0.93, 1.00) |
| Aged 19-24 | 1.04 (1.03, 1.06) | 0.98 (0.97, 1.00) | | 0.66 (0.59, 0.74) |
| **Sex** |  |  | |  |
| Male | 1 | 1 | | 1 |
| Female | 1.23 (1.16, 1.30) | 1.49 (1.42, 1.56) | | 1.85 (1.77, 1.93) |
| **IMD Quintile** | |  | |  |
| Q1: Most deprived 20% | 1.03 (0.95, 1.11) | 1.08 (1.01, 1.15) | | 0.98 (0.92, 1.04) |
| Q2 | 1.01 (0.93, 1.10) | 1.03 (0.96, 1.10) | | 0.98 (0.92, 1.05) |
| Q3 | 0.99 (0.91, 1.08) | 1.04 (0.97, 1.11) | | 1.05 (0.98, 1.12) |
| Q4 | 0.95 (0.87, 1.04) | 0.99 (0.92, 1.07) | | 1.00 (0.93, 1.07) |
| Q5: Least deprived 20% | 1 | 1 | | 1 |
| **Presence of different groups of chronic conditions (out of 7)** | | |  |  |
| None | 1 | 1 | | 1 |
| 1 | 1.71 (1.61, 1.82) | 1.52 (1.45, 1.60) | | 1.46 (1.40, 1.53) |
| 2 | 2.73 (2.52, 2.95) | 2.43 (2.29, 2.58) | | 1.96 (1.85, 2.08) |
| 3+ | 6.09 (5.53, 6.71) | 4.65 (4.32, 5.00) | | 3.53 (3.29, 3.79) |

**note that follow-up was available up to max of age 20 years old for the oldest children in the cohort (born in 1998)*

### Analyses focussing on young people with specific learning disability diagnosis or a diagnosis of “high-risk” condition

Appendix Table 20 – Adjusted hospital admission rates and rate ratios (95% confidence intervals)) for hospital admissions for young people with a specific learning disability diagnosis or a diagnosis of a “high-risk” condition

|  | **Planned admissions** | **Unplanned admissions** |
| --- | --- | --- |
| **Admission rates at baseline for each age group per 100 person-years (intercept)** | | |
| Age 10 | 7.6 (7.1, 8.2) | 2.6 (2.4, 2.8) |
| Age 16 | 7.9 (7.3, 8.6) | 3.3 (3.0, 3.6) |
| Age 19 | 4.8 (4.4, 5.2) | 3.6 (3.3, 3.9) |
| **All estimates below are rate ratios:** | |  |
| **Change in admission rate per year by age group (slope)** | | |
| Age 10-15 | 0.99 (0.98, 1.00) | 1.04 (1.03, 1.05) |
| Age 16-18 | 0.88 (0.86, 0.90) | 1.03 (1.01, 1.06) |
| Aged 19-24 | 0.99 (0.98, 1.00) | 1.04 (1.03, 1.05) |
| **Sex** |  |  |
| Male | 1 | 1 |
| Female | 0.85 (0.82, 0.88) | 1.00 (0.96, 1.04) |
| **IMD Quintile** |  |  |
| Q1: Most deprived 20% | 0.81 (0.76, 0.86) | 1.34 (1.26, 1.43) |
| Q2 | 0.88 (0.83, 0.94) | 1.19 (1.12, 1.28) |
| Q3 | 0.93 (0.87, 0.99) | 1.12 (1.05, 1.20) |
| Q4 | 0.99 (0.93, 1.06) | 1.06 (0.98, 1.13) |
| Q5: Least deprived 20% | 1 | 1 |
| **Year of birth** |  |  |
| 1990-1993 | 1.39 (1.33, 1.46) | 1.29 (1.22, 1.35) |
| 1994-1997 | 1.13 (1.08, 1.19) | 1.13 (1.08, 1.19) |
| 1998-2001 | 1 | 1 |
| **Presence of different groups of chronic conditions (out of 7)** | |  |
| None | 1 | 1 |
| 1 | 1.81 (1.71, 1.91) | 1.60 (1.51, 1.69) |
| 2 | 3.21 (3.03, 3.40) | 2.73 (2.57, 2.90) |
| 3+ | 8.99 (8.54, 9.46) | 8.02 (7.60, 8.47) |

*IMD=Index of Multiple Deprivation.
All estimates are mutually adjusted for covariates listed in the table.*

## Cross-sectional analyses of outpatient records

HES outpatient records are available since 1^st^ April 2003, therefore available follow-up (between 2004-2018) is not long enough to observe changes in outpatient care at ages 10-24 years old from a longitudinal study. Instead, we used data for children with learning disabilities or autism indicated in HES records, who were born in 1990-2008, for cross-sectional analyses of trends in outpatient appointments at ages 10-24 years old in 2014-2018.

We calculated the average number of appointments per child by age at appointment. We used the number of attended appointments as the numerator. The denominator was the population of children with learning disabilities or autism included in the HES study cohort, who were alive at each age per calendar year, summed over 2014-2018.

Appendix Figure 6 – Average number of outpatient appointments per child by age (cross-sectional analysis of data form 2014-2018)

# References

1. Sheehan R, Hassiotis A, Walters K, Osborn D, Strydom A, Horsfall L. Mental illness, challenging behaviour, and psychotropic drug prescribing in people with intellectual disability: UK population based cohort study. *BMJ*. 2015;351:h4326. doi:10.1136/bmj.h4326

2. British Medical Association (BMA), National Health Service (NHS) England. 2019/20 General Medical Services (GMS) contract Quality and Outcomes Framework (QOF). *Guid GMS Contract 2019/20*. 2019;April. Accessed August 18, 2020. https://www.england.nhs.uk/wp-content/uploads/2019/05/gms-contract-qof-guidance-april-2019.pdf

3. Department of Health. *A Simple Guide to Payment by Results*.; 2012. Accessed February 10, 2020. www.dh.gov.uk/pbr

4. Wijlaars LPMM, Hardelid P, Woodman J, Allister J, Cheung R, Gilbert R. Who comes back with what: a retrospective database study on reasons for emergency readmission to hospital in children and young people in England. *Arch Dis Child*. 2016;101(8):714-718. doi:10.1136/archdischild-2015-309290

5. NHS DIGITAL. HES Data Dictionary - Admitted Patient Care. *Hscic*. 2018;(April). Accessed November 16, 2019. https://datadictionary.ices.on.ca/Applications/DataDictionary/Default.aspx

6. Office for National Statistics. Analysis of population estimates tool. Office for National Statistics. Published 2020. Accessed May 4, 2021. https://www.ons.gov.uk/peoplepopulationandcommunity/populationandmigration/populationestimates/datasets/analysisofpopulationestimatestool

7. Office for National Statistics. Deaths by single year of age tables, UK. Published 2020. Accessed May 4, 2021. https://www.ons.gov.uk/peoplepopulationandcommunity/birthsdeathsandmarriages/deaths/datasets/deathregistrationssummarytablesenglandandwalesdeathsbysingleyearofagetables
